# Endogenous retroviruses promote homeostatic and inflammatory responses to the microbiota

**DOI:** 10.1016/j.cell.2021.05.020

**Published:** 2021-07-08

**Authors:** Djalma S. Lima-Junior, Siddharth R. Krishnamurthy, Nicolas Bouladoux, Nicholas Collins, Seong-Ji Han, Erin Y. Chen, Michael G. Constantinides, Verena M. Link, Ai Ing Lim, Michel Enamorado, Christophe Cataisson, Louis Gil, Indira Rao, Taylor K. Farley, Galina Koroleva, Jan Attig, Stuart H. Yuspa, Michael A. Fischbach, George Kassiotis, Yasmine Belkaid

**Affiliations:** 1 Metaorganism Immunity Section, Laboratory of Immune System Biology, National Institute of Allergy and Infectious Diseases, National Institutes of Health, Bethesda, MD 20892, USA; 2 NIAID Microbiome Program, National Institute of Allergy and Infectious Diseases, National Institutes of Health, Bethesda, MD 20892, USA; 3 Department of Bioengineering and ChEM-H, Stanford University, Stanford, CA 94305, USA; 4 NIH Center for Human Immunology, Bethesda, MD 20896, USA; 5 In Vitro Pathogenesis Section, Laboratory of Cancer Biology and Genetics, Center for Cancer Research, National Cancer Institute, National Institutes of Health, Bethesda, MD 20892, USA; 6 Immunology Graduate Group, University of Pennsylvania, Philadelphia, PA 19104, USA; 7 Kennedy Institute of Rheumatology, Nuffield Department of Orthopaedics, Rheumatology and Musculoskeletal Sciences, University of Oxford, Roosevelt Drive, Oxford OX3 7FY, UK; 8 Retroviral Immunology, The Francis Crick Institute, 1 Midland Road, London NW1 1AT, UK; 9 Department of Medicine, Faculty of Medicine, Imperial College London, London W2 1PG, UK

**Keywords:** Staphylococcus epidermidis, microbiota, skin immunity, tissue repair, endogenous retrovirus, high fat diet, keratinocytes, antiretroviral, STING, T cells

## Abstract

The microbiota plays a fundamental role in regulating host immunity. However, the processes involved in the initiation and regulation of immunity to the microbiota remain largely unknown. Here, we show that the skin microbiota promotes the discrete expression of defined endogenous retroviruses (ERVs). Keratinocyte-intrinsic responses to ERVs depended on cyclic GMP-AMP synthase (cGAS)/stimulator of interferon genes protein (STING) signaling and promoted the induction of commensal-specific T cells. Inhibition of ERV reverse transcription significantly impacted these responses, resulting in impaired immunity to the microbiota and its associated tissue repair function. Conversely, a lipid-enriched diet primed the skin for heightened ERV- expression in response to commensal colonization, leading to increased immune responses and tissue inflammation. Together, our results support the idea that the host may have co-opted its endogenous virome as a means to communicate with the exogenous microbiota, resulting in a multi-kingdom dialog that controls both tissue homeostasis and inflammation.

## Highlights

• Endogenous retrovirus sensing is required for commensal-specific immunity
• Reverse transcription inhibition impairs commensal-induced immunity in the skin
• cGAS/STING signaling in keratinocytes is required for skin immunity to the microbiota
• Enhanced endogenous retrovirus expression promotes microbiota-induced inflammation

## Graphical abstract

**Figure:**
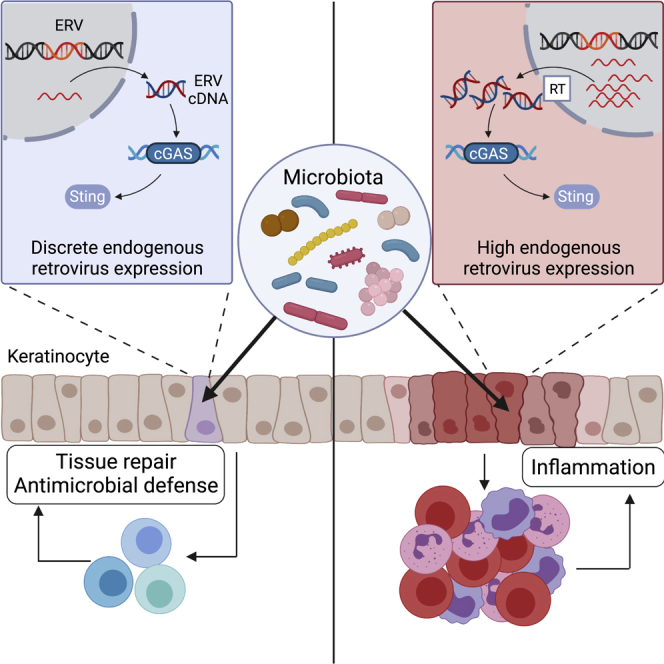

## Teaser

The host immune system uses the endogenous virome to communicate with the exogenous microbiota in a multi-kingdom interaction that modulates tissue repair or inflammation.

## Introduction

Metazoans exist as meta-organisms composed of both the host and its symbiotic microbiota (Sender et al., 2016). These complex communities of microbes broadly control host physiology, including numerous aspects of the immune system (Belkaid and Harrison, 2017). The immune system recognizes these resident microbes at all barrier surfaces; this response includes the induction of classical and non-classical T cell responses directed at the microbiota itself (Cong et al., 2009; Hand et al., 2012; Naik et al., 2015; Yang et al., 2014). What differentiates these responses from those resulting from pathogenic microbes is that both the initiation of immune responses and accumulation of commensal-specific lymphocytes within tissues occur in the absence of inflammation, a process referred to as homeostatic immunity (Belkaid and Harrison, 2017). Immunity to the microbiota controls numerous aspects of host physiology ranging from antimicrobial defense to tissue repair. Notably, exposure to defined members of the skin microbiota promotes the accumulation of various lymphocyte subsets within the skin that can promote tissue repair (Belkaid and Harrison, 2017; Constantinides et al., 2019; Linehan et al., 2018). Further, one of the dominant actions of these cells is mediated via the release of interleukin-17A (IL-17A), which can promote antimicrobial responses by keratinocytes (Constantinides et al., 2019; Naik et al., 2012, 2015).

Our current understanding of immunity postulates that the induction of immune responses requires inflammation or tissue damage (Matzinger, 2002; Murphy and Weaver, 2016). However, within this framework, it is unclear how the immune system actively recognizes and responds to the microbiota in the absence of classical inflammatory processes. More specifically, the factors responsible for recognizing commensal microbes discretely within tissues remain poorly understood. Based on the evolutionary alliance between the microbiota and the immune system, we hypothesized that such mechanisms may be highly ubiquitous and conserved.

Of particular interest, the microbiota promotes type I interferon (IFN-I) and antiviral states in tissues (Abt et al., 2012; Di Domizio et al., 2020; Gutierrez-Merino et al., 2020; Schaupp et al., 2020); from these findings, the microbiota is thought to protect the host against various viral infections (Abt et al., 2012; Bradley et al., 2019; Steed et al., 2017). Recent evidence also supports the idea that, within the gut, the microbiota promotes IFN-I production through viral sensors, including stimulator of interferon genes protein (STING) (Gutierrez-Merino et al., 2020). Together, these observations raise the intriguing possibility that responses to the microbiota may be entwined with responses to endogenous viral elements.

Retroviruses can integrate into the germline, thus becoming part of the host genome. As such, the long-standing symbiotic relationship between mammals and microbes encompasses not only those with exogenous microbes, such as bacteria, fungi, and viruses, but also endogenous retroviruses (ERVs) (Johnson, 2015). Most ERVs are inactive; however, several ERVs express intact open reading frames (ORFs). Numerous physiological and pathological processes rely on the transcriptional activity of ERVs (Stoye, 2012), and thus these retroelements are under constant immune pressure. In several contexts, microbial exposure can control ERV expression (Panova et al., 2020; Young et al., 2012a); germ-free mice lose basal intestinal expression of several ERV groups, and microbe-derived products, such as Toll-like receptor (TLR) ligands, drive transcription of defined ERVs (Young et al., 2014). However, whether ERVs directly contribute to the induction of responses to the microbiota remains unknown. Further, to what extent heightened expression of ERVs could, in defined settings, contribute to the inflammatory impact of the microbiota has not been addressed.

Here, we propose that the immune system may have co-opted its endogenous virome as a way to communicate with its exogenous microbiome. Notably, our results support the idea that the intensity and quality of immune responses to the microbiota under both steady-state and inflammatory settings are controlled by the expression of ERVs.

## Results

### Skin colonization by commensal bacteria promotes an antiviral signature

**Figure 1:**
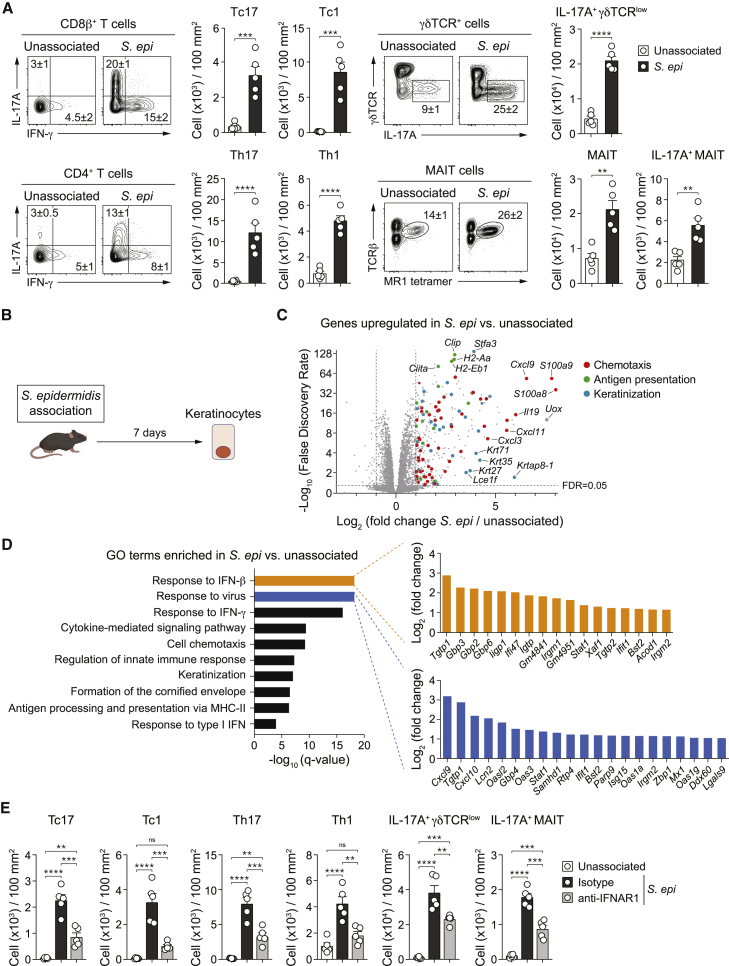
*S. epidermidis* colonization promotes an antiviral program, and responses to *S. epidermidis* are type I IFN dependent (A) Frequency and absolute number of IL-17A^+^ (Tc17) or IFN-γ^+^ (Tc1) CD8^+^ T cells, IL-17A^+^ (Th17) or IFN-γ^+^ (Th1) CD4^+^ T cells, and IL-17A^+^ γδTCR or MAIT cells in the skin of unassociated or *S. epidermidis* associated WT mice 14 days post-association. (B) Experimental schematic. (C) Volcano plot for log_2_ fold change in gene expression in CD49f^+^Sca-1^+^ keratinocytes isolated from *S. epidermidis* associated versus unassociated mice. Representative genes associated with chemotaxis (red), antigen presentation (green), and keratinization (blue) are highlighted. (D) Gene ontology (GO) assignments of top 10 GO terms that were enriched in Sca-1^+^CD49f^+^ keratinocytes from *S. epidermidis* associated versus unassociated WT mice. Upregulated genes are shown for specific pathways of interest. (E) Absolute number of indicated cell subsets in the skin of unassociated or *S. epidermidis* associated WT mice treated with anti-IFNAR1 neutralizing antibody (anti-IFNAR1) or isotype control. (A and E) ^∗^p < 0.05, ^∗∗^p <, 0.01, ^∗∗∗^p < 0.001, and ^∗∗∗∗^p < 0.0001 as calculated using a Student’s t test (A) or one-way ANOVA with Holm-Šidák multiple comparison test (E). Data are represented as mean ± SEM. In (A), numbers correspond to the frequency of the gated populations ± SEM. Data are representative of four (A) or two (C–E) independent experiments using 4 to 5 mice per group (with the dots in A and E each representing individual mice). See also Figure S1.

**Figure S1:**
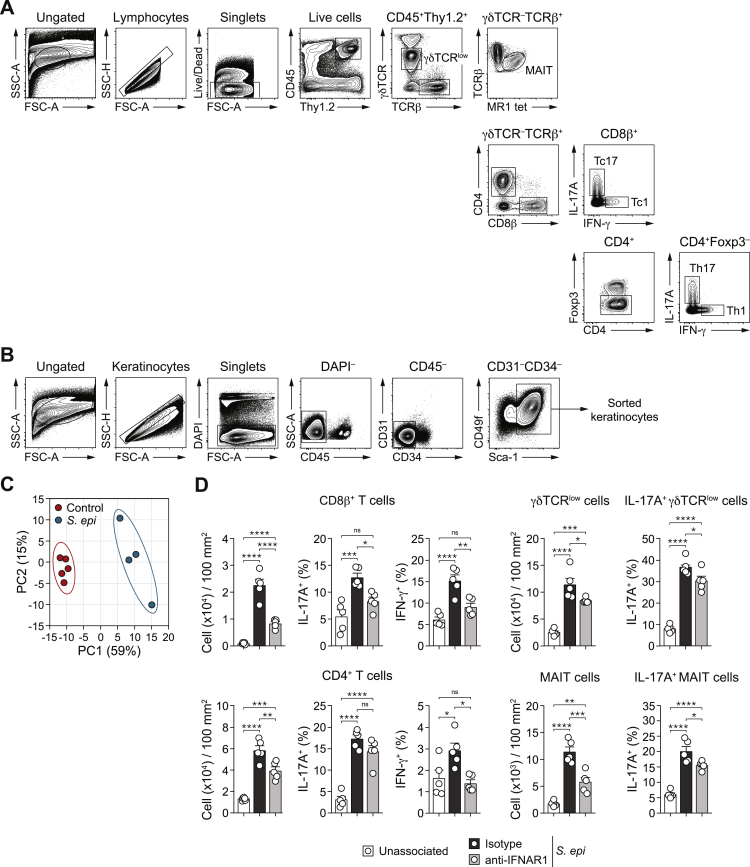
Type I IFN is required for *S. epidermidis* induced T cells responses in the skin, related to Figure 1 (A) Flow cytometry gating strategy used to identify various T cell subsets and MAIT cells. Tc1 and Tc17 cells are defined as live CD45^+^ Thy1.2^+^ TCRβ^+^ γδTCR^–^ CD8b^+^ IFN-γ^+^ and live CD45^+^ Thy1.2^+^ TCRβ^+^ γδTCR^–^ CD8b^+^ IL-17A^+^, respectively. Th1 and Th17 cells are defined as live CD45^+^ Thy1.2^+^ TCRβ^+^ γδTCR^–^ CD4^+^ Foxp3^–^ IFN-γ^+^and live CD45^+^ Thy1.2^+^ TCRβ^+^ γδTCR^–^ CD4^+^ Foxp3^–^ IL-17A^+^, respectively. γδTCR^low^ cells are defined as live CD45^+^ Thy1.2^+^ TCRβ^–^ γδTCR^low^. MAIT cells are defined as live CD45^+^ Thy1.2^+^ TCRβ^+^ MR1-tetramer^+^. (B) Flow cytometry gating strategy use to identify interfollicular keratinocytes (DAPI^–^ CD45^–^ CD31^–^ CD34^–^ CD49f^+^ Sca-1^+^) in single cells suspensions isolated from the epidermis of mouse ear pinnae 7 days post daily *S. epidermidis* association. (C) Principal component analysis of global gene expression of RNA-seq performed on interfollicular keratinocytes sorted from the epidermis at day 7 post *S. epidermidis* association. Ellipses denote 95% confidence intervals of the mean. Keratinocytes were isolated from ear pinnae. (D) Absolute number of CD8^+^ T cells, CD4^+^ T cells, γδTCR^low^ and MAIT cells, and frequency of IL-17A^+^ or IFN-γ^+^ CD8^+^ or CD4^+^ T cells, as well as IL-17A^+^γδTCR^low^ and MAIT cells in the ear pinnae of unassociated mice or *S. epidermidis*-associated mice treated with anti-IFNAR1 or isotype control antibodies. Cells were stimulated with PMA and ionomycin. ^∗^*P* ˂ 0.05, ^∗∗^*P* ˂ 0.01, ^∗∗∗^*P* ˂ 0.001, ^∗∗∗∗^*P* ˂ 0.0001 as calculated with one-way ANOVA with Holm-Šidák multiple comparison test. Data are represented as mean ± SEM. Data are representative of two independent experiments using 4-5 mice per group (with the symbols in (C) and (D) each representing an individual mouse).

Skin colonization with commensals promotes the accumulation of various T cell subsets within the skin in a manner uncoupled from inflammatory processes (Naik et al., 2012, 2015). Notably, skin colonization of specific pathogen-free (SPF) mice with *Staphylococcus epidermidis* induced a significant increase in both the numbers and frequencies of Tc17 (IL-17A producing CD8^+^ T cells), Tc1 (IFN-γ producing CD8^+^ T cells), Th17 (IL-17A producing CD4^+^ T cells), Th1 (IFN-γ producing CD4^+^ T cells), mucosal-associated invariant T (MAIT), and IL-17A producing γδ T cells detectable by day 5, peaking at 14 days post-association, and maintained for several months (Figures 1A and S1A; Constantinides et al., 2019; Linehan et al., 2018; Naik et al., 2015). Keratinocytes represent the primary interface between the host and its microbiota (Chen et al., 2018). To uncover upstream mechanisms responsible for the induction of homeostatic immunity to the microbiota, we characterized keratinocyte responses to *S. epidermidis* colonization prior to the peak of T cell responses (7 days post-association). The gene expression of interfollicular keratinocytes (Figure S1B) exposed to *S. epidermidis* was distinct and clustered separately from keratinocytes isolated from controls (Figure S1C). Gene Ontology (GO) pathways enriched in the transcriptome of purified keratinocytes from *S. epidermidis* colonized mice revealed upregulation of numerous genes linked to immune activation compared to keratinocytes from controls (Figures 1B and 1C). In agreement with enhanced T cell accumulation, keratinocytes from *S. epidermidis* colonized mice expressed elevated levels of chemokines (Figure 1C) associated with lymphocyte homing (Metzemaekers et al., 2018). Further, and as we have previously shown, colonization increased expression of genes implicated in antigen processing and presentation (Tamoutounour et al., 2019; Figures 1C and 1D). Of particular interest, and despite the lack of tissue inflammation, gene expression associated with IFN-I and antiviral responses were among the most upregulated within keratinocytes (Figure 1D). Notably, keratinocytes from colonized mice expressed higher transcript levels of numerous interferon-stimulated genes (ISGs) compared to keratinocytes from control mice (Figure 1D).

To assess whether IFN-I contributed to immune responses to *S. epidermidis*, mice were treated with a blocking antibody for the IFN-I receptor, interferon alpha receptor 1 (IFNAR1), or isotype control during colonization. Treatment with anti-IFNAR1 antibody significantly reduced the number of all T cell subsets induced by *S. epidermidis* as well as their ability to express IL-17A and IFN-γ, with the exception of IL-17A production by CD4^+^ T cells (Figures 1E and S1D). Thus, *S. epidermidis* association triggers an antiviral program in keratinocytes and IFN-I promotes T cell responses to this commensal.

### *S. epidermidis* association promotes transcription of endogenous retroelements

The antiviral signature induced by *S. epidermidis* colonization supported the idea that response to skin commensals may be associated with the sensing of endogenous viral elements. The mammalian genome contains numerous and diverse endogenous retroelements (Kassiotis and Stoye, 2016), the activation of which is potentially sensed by the immune system during microbial colonization. Relevant to our observation, the induction of ERVs has been previously associated with the induction of IFN-I responses (Kassiotis and Stoye, 2016). Among other groups, these retroelements include two distinct groups in terms of phylogeny and replication cycle: ERVs, which are derived from ancestral integrations of exogenous retroviruses into the germline and long interspersed nuclear elements (LINEs) that are conserved in all vertebrates but highly expanded in mammals, including humans (Johnson, 2015).

**Figure 2:**
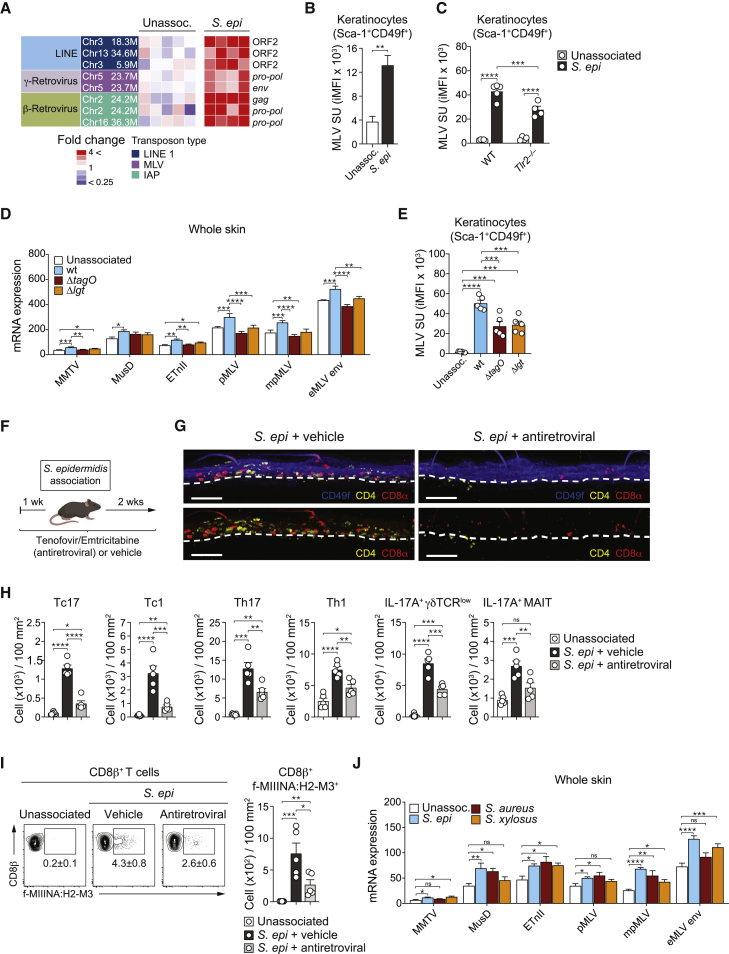
*S. epidermidis* promotes retroelements expression by keratinocytes (A) Heatmap displaying fold change of differentially expressed retroelement loci from RNA sequencing of Sca-1^+^CD49f^+^ keratinocytes from unassociated (unassoc.) or *S. epidermidis* (*S. epi*) associated mice at day 7 post-association. (B) MLV SU expression detected by flow cytometry on the surface of Sca-1^+^CD49f^+^ keratinocytes from unassociated or *S. epi* associated mice at day 7 post-association. Integrated MFI (iMFI) of MLV SU expression is shown. (C) iMFI of MLV SU expression by Sca-1^+^CD49f^+^ keratinocytes from unassoc. or *S. epi* associated WT and *Tlr2*^*−/−*^ mice at day 7 post-association. (D and E) WT mice were associated for 7 days with wild type, *ΔtagO*, or *Δlgt S. epi* or left unassociated. (D) Transcript levels of the indicated ERVs measured in the ear pinnae by qRT-PCR. ERVs expression was normalized to *Gapdh* mRNA levels in the same sample. (E) iMFI of MLV SU expression by Sca-1^+^CD49f^+^ keratinocytes. (F) WT mice were treated with vehicle control (vehicle) or antiretroviral by oral gavage, beginning at 1 week before *S. epi* association for a total of 3 weeks. (G) Representative confocal microscopy images of whole-mount ear pinnae of WT mice treated with vehicle (*S. epi* + vehicle) or antiretroviral (*S. epi* + antiretroviral) stained for CD49f (blue), CD4 (yellow), and CD8α (red) at day 14 post-association. Scale bars represent 100 μm. (H) Absolute number of indicated T cell subsets at 14 days post-association from the skin of WT mice treated with either vehicle (*S. epi +* vehicle) or antiretroviral (*S. epi* + antiretroviral). (I) Frequency and absolute number of f-MIIINA:H2-M3 tetramer-positive CD8^+^ T cells from ear pinnae of unassoc. or *S. epi* associated mice treated with vehicle or antiretroviral. (J) Transcript levels of the indicated ERVs measured by qRT-PCR in the ear pinnae of mice after daily association with *S. epi*, *S. aureus*, or *S. xylosus* for 7 days. ERVs expression levels were normalized to *Gapdh* mRNA levels in the same sample. ^∗^p < 0.05, ^∗∗^p < 0.01, ^∗∗∗^p < 0.001, and ^∗∗∗∗^p < 0.0001 as calculated with a Student’s t test (B), one-way ANOVA (E, H, and I), or two-way ANOVA (C, D, and J) with Holm-Šidák multiple comparison test. Data are represented as mean ± SEM. Data are representative of two (A, C–E, G, I, and J) or three (B and H) independent experiments using 4 to 5 mice per group. Each dot represents an individual mouse. See also Figure S2.

**Figure S2:**
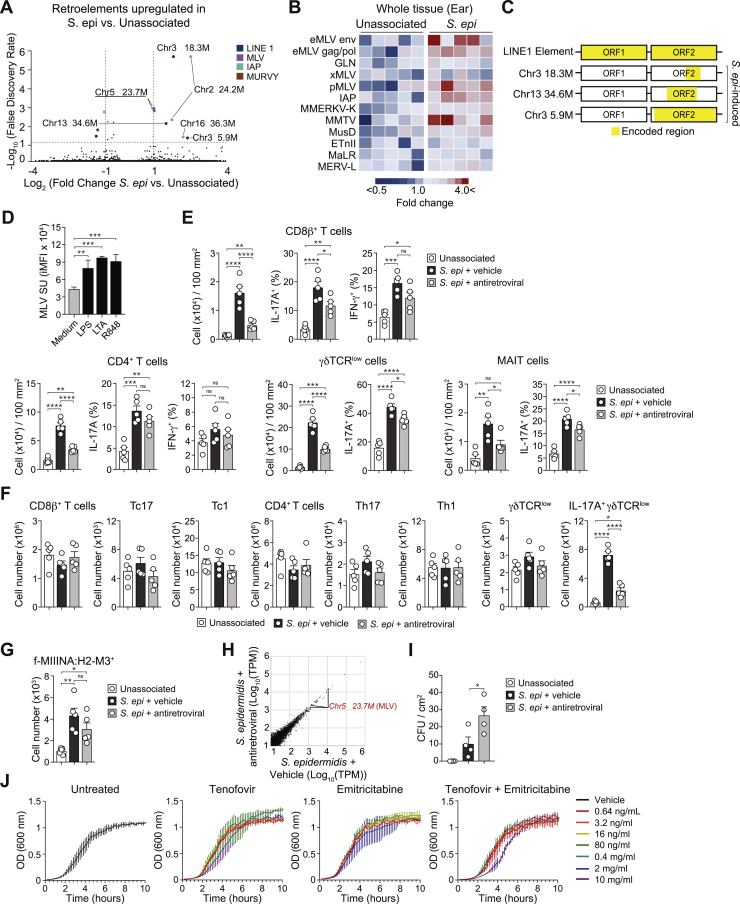
Endogenous retrovirus activity promotes the accumulation of T cells in the skin following *S. epidermidis* colonization, related to Figure 2 (A) Volcano plot of expressed loci from sorted Sca-1^+^CD49f^+^ keratinocytes from unassociated or *S. epidermidis*-associated at day 7 post-association. Underlined denotes locus containing an active reverse transcriptase. (B) Heatmap showing transcript levels of the indicated ERVs measured by qRT-PCR of mRNA isolated from ear pinnae from unassociated or daily *S. epidermidis*-associated mice at day 7 post association. Values were normalized to *Gapdh* expression in the same sample. (C) Schematic showing encoded regions of LINE-1 elements in the C57BL/6J (WT) mice genome. Regions highlighted in yellow represent the nucleotide regions encoded in the given locus. (D) Calculated iMFI of MLV SU expression detected by flow cytometry on the surface of CD49f^+^Sca-1^+^ primary keratinocytes isolated from neonatal mice stimulated with TLR agonists. (E-G) WT mice were treated daily with either vehicle control (*S. epi +* vehicle) or antiretroviral (*S. epi* + antiretroviral) beginning at 1 week before *S. epidermidis* association for a total of 3 weeks. Two weeks post association, T cell populations were evaluated by flow cytometry. (E and F) Absolute number of CD8^+^ T cells, CD4^+^ T cells, γδTCR^low^ T cells, IL-17A^+^ (Tc17) or IFN-γ^+^ (Tc1) CD8^+^ T cells, IL-17A^+^ (Th17) or IFN-γ^+^ (Th1) CD4^+^ T cells, or IL-17A^+^ γδTCR^low^ T cells (E) in the ear pinnae or (F) the spleen. (G) Absolute number of bead-enriched f-MIIINA:H2-M3 tetramer positive CD8β^+^ T cells in the spleen. Data are represented as mean ± SEM. Each dot represents an individual mouse. (H) WT mice were treated daily with either vehicle control (*S. epi* + vehicle) or antiretroviral (*S. e*pi + antiretroviral) beginning 1 week before daily *S. epidermidis* association for a total of 2 weeks. Seven days post-association, RNA was purified from sorted Sca-1^+^CD49f^+^ keratinocytes and sequenced. The expression of the *S. epidermidis-*induced ERV, Chr5 23.7M is highlighted in red. (I) Enumeration of *S. epidermidis* colony-forming units (CFU) at day 14 post association in WT mice treated daily with either vehicle control (*S. epi* + vehicle) or antiretroviral (*S. epi* + antiretroviral) beginning at 1 week before *S. epidermidis* association. (J) Growth curve (OD600) of *S. epidermidis* grown in media treated with vehicle, tenofovir and emtricitabine alone or with a combination of tenofovir and emtricitabine at different concentrations. ^∗^*P* ˂ 0.05, ^∗∗^*P* ˂ 0.01, ^∗∗∗^*P* ˂ 0.001, ^∗∗∗∗^*P* ˂ 0.0001 as calculated with one-way ANOVA with Holm-Šidák multiple comparison test (C, E-H). Data are representative of two (A-D, H-J) or three (E) and (F) independent experiments using 4-5 mice per group (A), (B), E-I). Graphs in (C) and (J) represent the average of a technical triplicate.

Skin association with *S. epidermidis* promoted the expression of several families of endogenous retroelements within the skin (Table 1; Figure 2A). Increased expression of retroelement genes from three major families of endogenous retroelements was also detected in purified keratinocytes: the LINE-1 elements; the intracisternal alpha particle (IAP) elements (an ERV family related to betaretroviruses); and one locus, Chr5 23.7M (also known as Xmv45; Table 1), from the murine leukemia virus (MLV) group, an ERV family related to the extant gammaretrovirus murine leukemia virus (Table 1; Figures 2A, S2A, and S2B).

**Table 1:** Chromosomal location and common names of labeled endogenous retroelements based on GRCm38

Nomenclature used in current publication	Endogenous retrovirus gene locations (strand)			LINE-1 gene locations (strand)	Other names for locus
	*gag*	*pol*	*env*	ORF2	
Chr1 69.5M		chr1:6969944569702763 (−)			
Chr1 182.3M	chr1:182258186-182259898 (+)	chr1:182259902-182263444 (+)	chr1:182263297-182265366 (+)		
Chr2 16M	chr2:16026714-16028352 (−)	chr2:16023111-16026710 (−)	chr2:16021273-16023315 (−)		
Chr2 24.2M	chr2:24193743-24195578 (+)	chr2:24195434-24197905 (+)			
Chr5 110M	chr5:109905189-109906993 (−)	chr5:109901775-109905185 (−)			
Chr5 23.7M	chr5:23706608-23708539 (−)	chr5:23703026-23706604 (−)	chr5:23701149-23703218 (−)		Xmv45
Chr5 24.2M	chr5:24216220-24217932 (+)	chr5:24217932-24219533 (+)	chr5:24219518-24221581 (+)		
Chr5 25.2M	chr5:25231883-25233520 (+)	chr5:25233524-25236934 (+)	chr5:25236919-25238892 (+)		Mpmv13
Chr6 73.3M	chr6:73291329-73292966 (−)	chr6:73287915-73290254 (−)			
Chr7 29.6M	chr7:29616273-29617985 (+)	chr7:29617989-29621588 (+)	chr7:29621384-29623171 (+)		
Chr7 30.6M	chr7:30689706-30691418 (+)	chr7:30691422-30694964 (+)	chr7:30694817-30696886 (+)		Pmv15
Chr8 123.2M	chr8:123165736-123167538 (+)	chr8:123167542-123171295 (+)			
Chr8 123.4M	chr8:123431904-123433835 (−)	chr8:123428313-123431900 (−)	chr8:123426364-123428661 (−)		Emv2
Chr8 85.1M	chr8:85129579-85131291 (−)	chr8:85126740-85129583 (−)	chr8:85126466-85126753 (−)		
Chr9 62.4M	chr9:62440115-62441827 (+)	chr9:62441827-62445417 (+)	chr9:62445225-62447282 (+)		
Chr11 88.9M	chr11:88894192-88895901 (−)	chr11:88892779-88894188 (−)	chr11:88890914-88892971 (−)		
Chr13 21.8M	chr13:21813594-21815231 (+)	chr13:21815235-21818834 (+)			
Chr13 99M	chr13:98992477-98994189 (−)	chr13:98988931-98992473 (−)	chr13:98987009-98989078 (−)		
Chr16 36.3M		chr16:36327413-36330097 (+)			
Chr16 93.7M	chr16:93703659-93705161 (−)	chr16:93700056-93703655 (−)	chr16:93698191-93700260 (−)		
Chr18 82.7M	chr18:82694617-82696228 (+)	chr18:82696232-82699831 (+)	chr18:82699627-82701696 (+)		Pmv20
Chr19 38.4M	chr19:3837690138377800 (+)	chr19:3837780438381094 (+)			
ChrY 4.8M	chrY:4801494-4802729 (−)	chrY:4797903-4801490 (−)	chrY:4796026-4798095 (−)		
Chr3 5.9M				chr3:5860754-5862853 (+)	
Chr3 18.3M				chr3:18293034-18293999 (−)	
Chr13 34.6M				chr13:34614319-34616805 (+)	

Retroelement nomenclature is based on the chromosomal location and strand designation GRCm38. The coordinates of genomic regions as encoding retroelements, as well as the retroelement gene that a given region encodes, were defined based on the gEVE database (Nakagawa and Takahashi, 2016).

We next tested the possibility that ERVs could coordinate responses to the microbiota via engagement of nucleic acid innate sensors. Retroelements can produce several molecular patterns, including double-stranded RNA (dsRNA) produced by abortive transcription (Chiappinelli et al., 2015; Gringhuis et al., 2017) and complementary DNA (cDNA) produced by reverse transcription (Gao et al., 2013; Kassiotis and Stoye, 2016). Increased expression of retroelement loci can create enhanced levels of dsRNA molecules; however, to increase cDNA levels, induced retroelement loci must encode functional copies of the genes required for reverse transcription. For example, LINE-1 elements require their encoded RNA chaperone (ORF1) and reverse transcriptase (ORF2) to be intact to reverse transcribe their mRNA molecules in *cis* (Martin and Bushman, 2001) and ERVs require the viral protease/polymerase polyprotein (*pro-pol*) gene to be functional in order to reverse transcribe (Hughes, 2015). We next assessed whether any retroelement loci upregulated by *S. epidermidis* colonization carried functional copies of these genes. We examined the expression of the distinct retroelement ORFs separately as a way to broadly assess the genomic integrity of each upregulated retroelement.

Although both ORF1 and ORF2 are required for retrotransposition, the LINE-1 elements upregulated by *S. epidermidis* either contained a truncated ORF2 or lacked ORF1 (Figure S2C). All three ERV loci induced by *S. epidermidis* encoded and transcribed the *pro-pol* ORF from their genomes; however, only the Chr5 23.7M locus contained a functional *gag* gene that was in frame with the *pro-pol* gene as well as a *pro-pol* gene that was not truncated (Table 1). In line with these observations, keratinocytes from mice associated with *S. epidermidis* also expressed higher levels of MLV surface glycoprotein (MLV SU) (Figure 2B). This supported the idea that MLV-derived ERVs may contribute to cDNA-induced innate responses in the context of skin microbial colonization.

We previously showed that the ability of *S. epidermidis* to induce CD8^+^ T cells was partially dependent on bacterial cell envelope teichoic acid and lipoproteins and host TLR2 expression (Chen et al., 2019). Microbial products and, in particular, TLR ligands can promote the expression of retroviral elements by various cells, including macrophages and B cells (Young et al., 2012a). To assess whether such a phenomenon was also true for keratinocytes, primary keratinocytes were exposed *in vitro* to various TLR agonists and MLV surface expression of the glycoprotein (MLV SU) on keratinocytes was measured. All TLR ligands tested, including ligands for TLR4 (lipopolysaccharide [LPS]), TLR2 (lipotheicoic acid [LTA]), and TLR7/8 (resiquimod [R848]), significantly enhanced the level of MLV SU expression by keratinocytes (Figure S2D). Further, increased MLV SU expression by keratinocytes following *S. epidermidis* association was reduced in TLR2-deficient mice, supporting the idea that TLR2 signaling contributes, at least in part, to ERV induction in the skin (Figure 2C).

*S. epidermidis* teichoic acid (*ΔtagO*) mutant was unable to induce any assessed ERV family (Figure 2D) following association, and the lipoprotein (*Δlgt*) mutant still induced MMTV and ETnII ERVs but was unable to induce any other ERV families assessed (Figure 2D). In agreement, the level of expression of MLV SU glycoprotein by keratinocytes following association with each mutant was significantly reduced compared to wild-type (WT) bacteria (Figure 2E). Taken together, these data support the notion that *S. epidermidis* wall teichoic acids and lipoproteins promote the induction of ERVs in the skin.

### Reverse transcription promotes T cell responses to the skin microbiota

We next assessed whether cDNA synthesis of endogenous retroelements controlled T cell responses to *S. epidermidis*. Mice were treated with a combination of antiretrovirals, tenofovir and emtricitabine, which are nucleotide and nucleoside reverse transcriptase inhibitors, respectively, or vehicle control prior and during association (Figure 2F). These compounds prematurely terminate nascent cDNA synthesis during reverse transcription (Banuelos-Sanchez et al., 2019). Imaging of skin revealed that antiretroviral treatment profoundly inhibited T cell accumulation within the epidermis following *S. epidermidis* association (Figure 2G). Antiretroviral treatment significantly reduced the absolute number of *S. epidermidis* specific CD8^+^ T cells (f-MIIINA:H2-M3^+^; Linehan et al., 2018) induced by *S. epidermidis* within the skin (Figure 2I), but not in the spleen (Figure S2G). Antiretroviral treatment also significantly decreased the absolute number of all T cell subsets induced by *S. epidermidis* association and significantly reduced IL-17 expression by CD8^+^, MAIT, and γδ T cells within the skin but did not impact T cells in secondary lymphoid organs (except γδ T cells; Figures 2H, S2E, and S2F). Antiretroviral treatment had no impact on ERV expression, supporting the idea that the impact of this treatment was associated with reduced responses to cDNA rather than changes in ERV mRNA levels (Figure S2H).

Of note, the genome of *S. epidermidis* did not contain homologs of the conventional bacterial reverse transcriptases ltrA and RT-Cas1 (Matsuura et al., 1997; Silas et al., 2016). Antiretrovirals did not impact the growth rate or overall growth yield of *S. epidermidis in vitro* and did not impair the ability of *S. epidermidis* to colonize the skin (Figures S2I and S2J). Thus, host reverse transcriptase activity is required to promote cognate responses to *S. epidermidis* in the skin. Further, other common skin commensals, such as *Staphylococcus aureus* and *Staphylococcus xylosus* (Chen et al., 2018), also promoted ERV expression within the skin (Figure 2J), supporting the idea that expression of retroelements could represent a general response to microbiota colonization.

### Antiretroviral treatment impacts keratinocyte responses to microbial colonization and associated tissue repair

**Figure S3:**
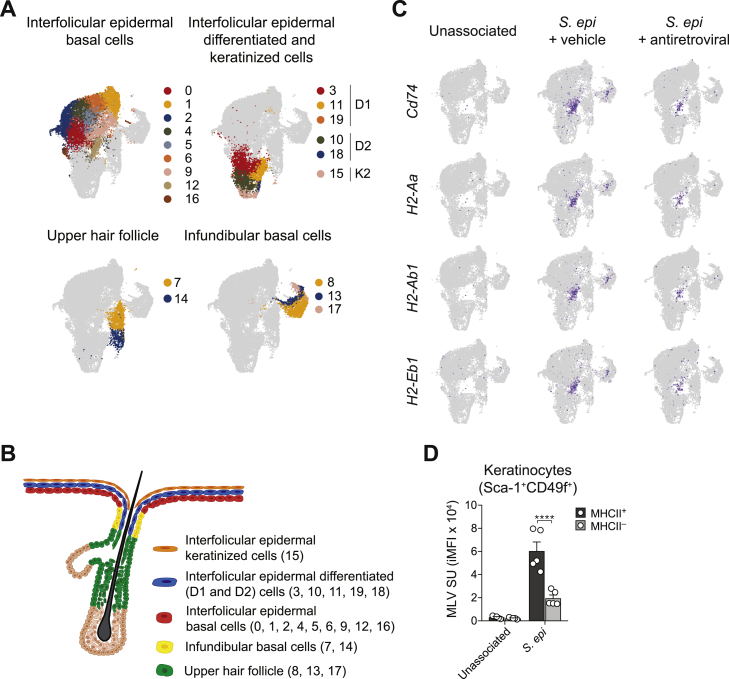
Single-cell RNA-seq analysis of interfollicular keratinocytes, related to Figure 3 WT mice were treated daily with either vehicle control (*S. epi +* vehicle) or antiretroviral (*S. epi* + antiretroviral) beginning at 1 week before *S. epidermidis* association for a total of 3 weeks. At day 14 post association, interfollicular keratinocytes were sorted by FACS from the epidermis of ear pinnae and analyzed by single-cell RNA-seq. (A) UMAP projection plots showing the expression profiles of keratinocytes. Colors represent cells clustered together based on similarity of global gene expression. Cells identity was assigned based on the expression level of specific genes: Interfollicular epidermal basal cells: *Krt14*^*hi*^*Mt2*^*hi*^*Mt1*^*hi*^*Postn*^*low*^; Interfollicular epidermal differentiated cells subcluster 1 (D1): *Krt14*^*mid*^*Krt10*^*mid*^*Mt4*^*hi*^; Interfollicular epidermal differentiated cells subcluster 2 (D2): *Krt14*^*low*^*Krt10*^*hi*^*Sbsn*^*hi*^;Interfollicular epidermal keratinized cells subcluster 2 (K2): *Krt14*^*low*^*Krt10*^*hi*^*Lor*^*hi*^*Flg2*^*hi*^; Upper hair follicle*: Krt79*^*hi*^*Krt17*^*hi*^*Defb*^*hi*^; and Infundibular basal cells: *Krt14*^*mid*^*Mt2*^*mid*^*Mt1*^*mid*^*Postn*^*+*^. (B) Schematic illustrating the anatomical localization of distinct populations of keratinocytes. (C) UMAP projection plots depicting expression of the indicated genes that are involved in antigen presentation on MHC class II. Data are representative of one experiment using 5 mice per group. (D) MLV SU expression (shown as integrated MFI) detected by flow cytometry on the surface of Sca-1^+^CD49f^+^MHCII^+^ and Sca-1^+^CD49f^+^MHCII^–^ keratinocytes from unassociated or *S. epidermidis*-associated mice at day 7 post-association. ^∗∗∗∗^*P* ˂ 0.0001 as calculated with one-way ANOVA with Holm-Šidák multiple comparison test (D). Data are representative of one (A and C) or three (D) independent experiments using 5 mice per group.

**Figure 3:**
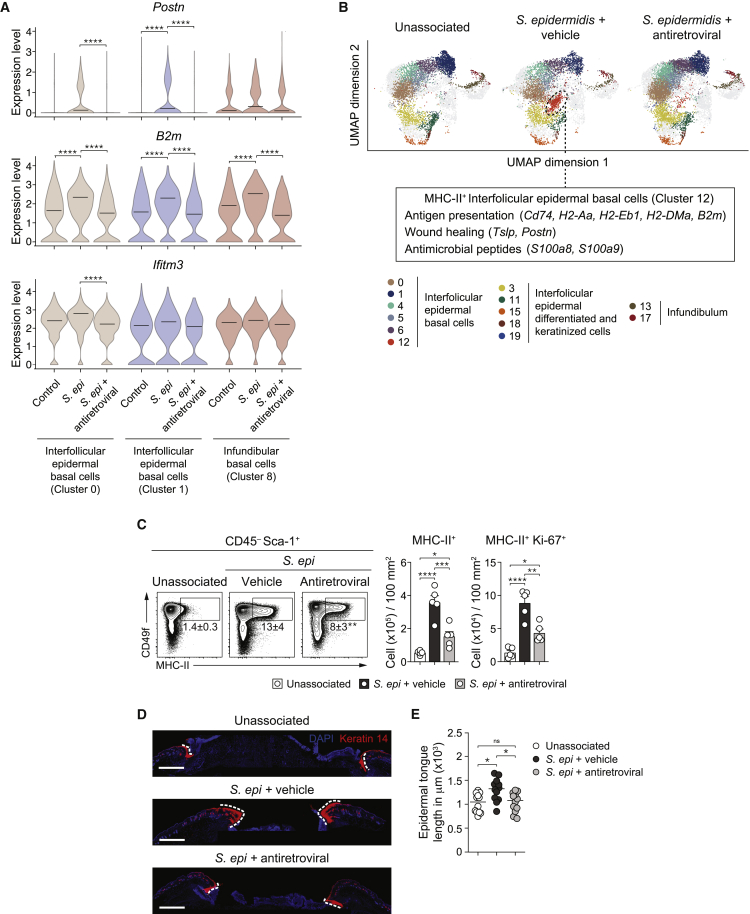
Antiretroviral treatment impairs keratinocyte responses to *S. epi* and tissue repair WT mice were treated with vehicle control (*S. epi +* vehicle) or antiretroviral (*S. epi* + antiretroviral) beginning 1 week before *S. epi* association for a total of 3 weeks. 2 weeks post-association, keratinocytes responses were analyzed. (A) scRNA-seq data from sorted keratinocytes showing the expression of *Postn*, *B2m*, and *Ifitm3* in individual keratinocytes for the clusters 0, 1 (interfollicular epidermal basal cells), and 8 (infundibular basal cells). (B) Uniform manifold approximation and projection (UMAP) plot displaying the distribution of the differentially abundant keratinocyte populations. Genes defining cluster 12 (MHCII^+^ keratinocytes) are denoted. (C) Flow cytometry analysis of MHC-II expression and Ki-67 co-expression in keratinocytes at 14 days post-association from *S. epi* associated WT mice treated with either vehicle (vehicle *+ S. epi*) or antiretroviral (*S. epi* + antiretroviral). Plots are gated on live CD45^−^CD31^−^CD34^−^Sca-1^+^. ^∗^ in the flow plots indicates significant difference between vehicle and antiretroviral treated group. (D and E) WT mice were treated with antiretroviral and associated with *S. epi*, followed by a back skin punch biopsy 12 days post-association. (D) Representative immunofluorescence images of wounds at day 5 after punch biopsy. Tissue sections are stained for basal keratinocytes (keratin 14 in red) and co-stained with DAPI (blue). Demarcated white dashed lines represent the epidermal tongue length during re-epithelization of the wounds. Scale bars represent 1,000 μm. (E) Quantification of the epidermal tongue length at day 5 post-wounding, with each dot representing the measured length of an individual epidermal tongue. ^∗^p < 0.05, ^∗∗^p < 0.01, ^∗∗∗^p < 0.001, and ^∗∗∗∗^p < 0.0001 as calculated with one-way ANOVA with Holm-Šidák multiple comparison test. Data are represented as mean ± SEM. Data are representative of one (A and B), two (D and E), or three (C) independent experiments using 4 to 5 mice per group. Each dot represents an individual mouse. See also Figure S3.

We have previously shown that T cell responses to the microbiota promotes several aspects of tissue physiology, including wound healing, via, in part, their action on keratinocytes (Harrison et al., 2019; Linehan et al., 2018). As such, we assessed the impact of antiretrovirals on keratinocyte responses at the peak of T cell responses to *S. epidermidis* via single-cell RNA sequencing (scRNA-seq) (Figures S3A and S3B). *S. epidermidis* induced expression of genes associated with wound healing, antigen presentation on major histocompatibility complex (MHC) class I, and antiviral defense (Ifitm3) in clusters from the interfollicular epidermal basal layer that were significantly decreased by antiretroviral treatment (Figure 3A). T cell responses to commensals promotes the development of organized cellular clusters within the epidermis that are surrounded by keratinocytes expressing a discrete program associated with antigen presentation, including high level of MHC class II (MHC-II), and antimicrobial defense (Tamoutounour et al., 2019). MHC-II^+^ keratinocytes display enhanced proliferation rates and higher levels of activation than MHC-II^−^ keratinocytes following *S. epidermidis* association (Tamoutounour et al., 2019). In agreement, genes linked with antigen presentation via MHC-II that are expressed in defined clusters of interfollicular epidermal and infundibular basal keratinocytes were significantly decreased by antiretroviral treatment (Figures S3A and S3C). More particularly, one cluster (cluster 12), enriched in genes associated with MHC-I and MHC-II antigen presentation, antimicrobial peptides, and wound healing, was only observed in *S. epidermidis* associated mice and was the most significantly decreased following antiretroviral treatment (Figures 3B and S3C). In line with the concept that MHC-II-expressing keratinocytes may represent “hot spots” for microbiota-induced T cells responses, MHC-II^+^ keratinocytes expressed higher level of MLV SU than MHC-II^−^ keratinocytes following commensal colonization (Figure S3D). The impact of antiretroviral treatment on keratinocytes was further confirmed by their decreased proliferation and expression of MHC-II following *S. epidermidis* association compared to controls (Figure 3C). Thus, antiretroviral treatment, via its action on T cell responses and/or keratinocytes, impacted the ability of *S. epidermidis* to promote a gene expression program consistent with antimicrobial defense and tissue repair.

Naive and *S. epidermidis* colonized mice, treated or not with antiretroviral drugs, were subjected to back-skin punch biopsy, and 5 days later, the epidermal tongue of proliferating keratinocytes was measured (Linehan et al., 2018). In line with reduced T cell responses, antiretrovirals significantly impaired the ability of *S. epidermidis* to promote wound healing compared to vehicle-treated mice (Figures 3D and 3E). Thus, inhibition of host reverse transcriptase has a broad impact on T cell responses to *S. epidermidis* and functionally inhibited homeostatic immunity to the microbiota.

### cGAS-STING signaling in keratinocytes is required for the induction of commensal-specific T cell responses

**Figure S4:**
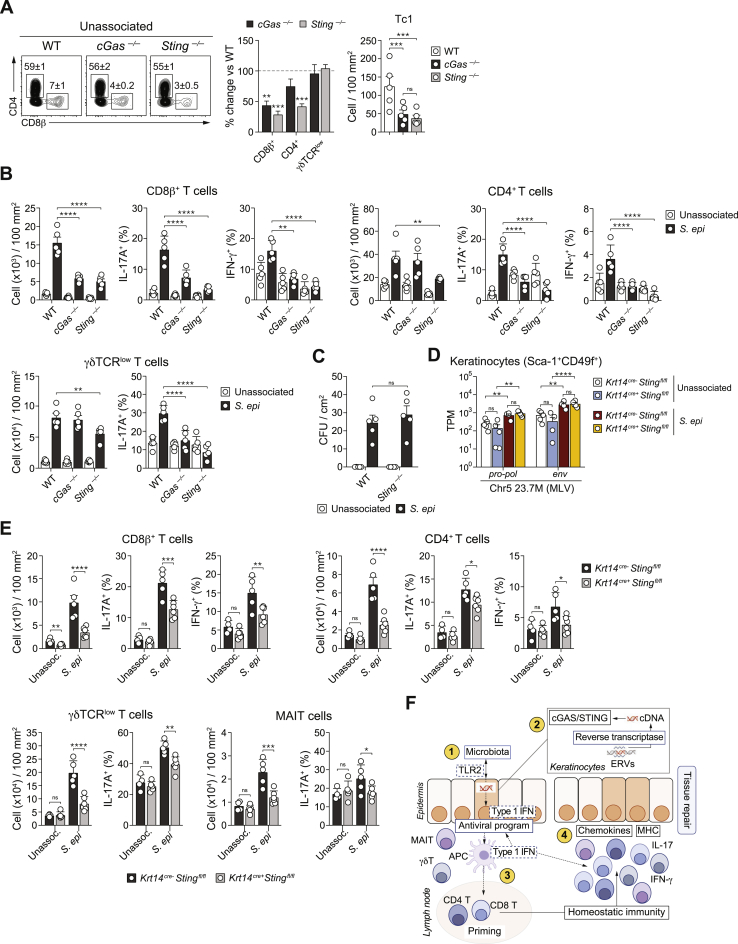
cGAS-STING signaling promotes T cells accumulation in the skin in response to *S. epidermidis* colonization, related to Figure 4 (A) Frequency of CD8^+^ and CD4^+^ (left) T cells, percentage change of the absolute numbers of CD8^+^, CD4^+^, γδTCR^low^ T cells (middle) and absolute number of IFN-γ^+^ CD8^+^ T cells (Tc1) (right) in the ear pinnae of naive (unassociated) WT, *cGas*^*–/–*^ and *Sting*^*–/–*^ mice. (B) Absolute number of CD8^+^, CD4^+^, γδTCR^low^ T cells and/or MAIT cells and frequency of IL-17A^+^ or IFN-γ^+^ CD8^+^ or CD4^+^ T cells and IL-17A^+^ γδTCR^low^ or MAIT cells in the ear pinnae from WT, *cGas*^*–/–*^ and *Sting*^*–/–*^mice associated with *S. epidermidis* (day 14) or left unassociated. (C) *S. epidermidis* CFU enumeration at 14 days post-association in WT and *Sting*^*–/–*^ mice topically associated with *S. epidermidis* (*S. epi*) or left unassociated. (D) Expression of Chr5 23.7 locus determined by RNASeq in Sca-1^+^CD49f^+^ keratinocytes purified from unassociated or daily *S. epi*-associated *Krt14*^*cre-*^*Sting*^*flox/flox*^ and *Krt14*^*cre+*^*Sting*^*flox/flox*^ mice at day 7 post-association. ^∗∗^ FDR < 0.01, ^∗∗∗∗^ FDR < 0.001. (E) Absolute number of CD8^+^, CD4^+^, γδTCR^low^ T cells and/or MAIT cells and frequency of IL-17A^+^ or IFN-γ^+^ CD8^+^ or CD4^+^ T cells and IL-17A^+^ γδTCR^low^ or MAIT cells in the ear pinnae from *Krt14*^*cre-*^*Sting*^*flox/flox*^ and *Krt14*^*cre+*^*Sting*^*flox/flox*^ mice associated with *S. epidermidis* (day 14) or left unassociated. (F) Proposed model for ERV control of T cell responses to the microbiota: 1- Discrete sensing of microbiota by keratinocytes (via, in part, TLR2-specific ligands) promotes the expression of defined ERVs; 2- Reverse transcription of ERVs leads to cytosolic accumulation of ERV-derived cDNAs that are sensed by the cGAS/STING pathway. Resulting activation of keratinocytes is associated with an antiviral program and type I IFN production; 3- Discrete keratinocyte “hot spots” could promote an environment favorable to the capture of microbiota-derived antigens by DCs and subsequent migration of these cells to the lymph node; 4- commensal-specific T cells migrate back to the skin where their accumulation and function could be promoted by ERV-activated keratinocytes. In the context of non-classical T cells such as MAIT cells, local responses to ERVs may be sufficient to control their local proliferation and accumulation in the skin. The result of this sequence of events is the accumulation of a network of commensal specific T cells able to broadly promote tissue physiology including tissue repair. All cells were stimulated with PMA and ionomycin. Each dot represents an individual mouse. ^∗^*P* < 0.05, ^∗∗^*P* < 0.01, ^∗∗∗^*P* < 0.001, ^∗∗∗∗^*P* < 0.0001 as calculated with two-way ANOVA with Holm-Šidák multiple comparison test. Data are represented as mean ± SEM. Data are representative of two independent experiments using 4-6 mice per group.

**Figure 4:**
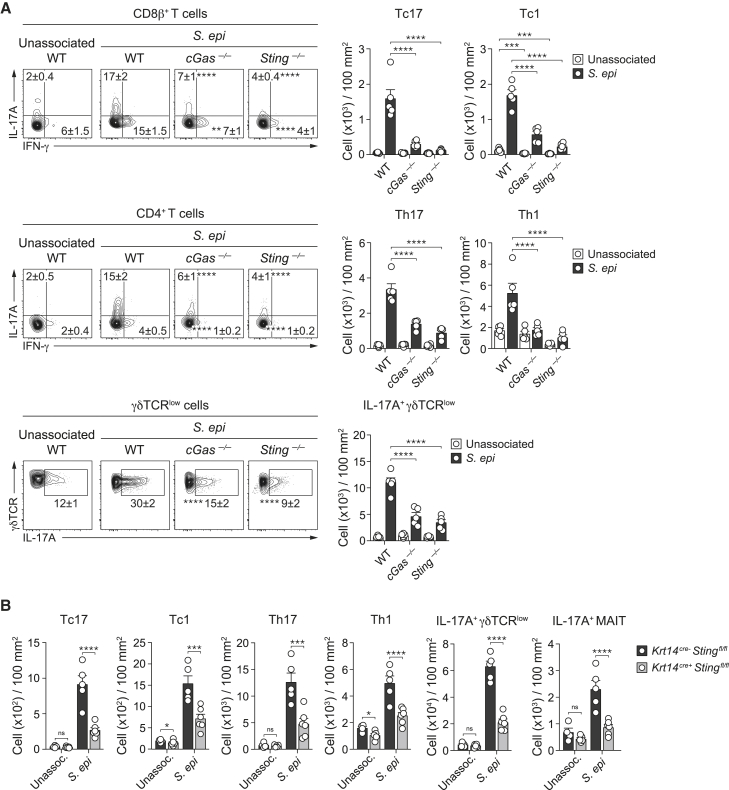
cGAS-STING signaling pathway is required for *S. epidermidis* induced T cells response WT, *cGas*^*−/−*^, *Sting*^*−/−*^, *Krt14*^*Cre-*^*Sting*^*flox/flox*^, and *Krt14*^*Cre+*^*Sting*^*flox/flox*^ mice were topically associated with *S. epi* or left unassociated. Two weeks post-association, T cell populations were evaluated by flow cytometry. Frequency and/or absolute number of indicated lymphocyte subsets in the ear pinnae from (A) WT, *cGas*^*−/−*^, and *Sting*^*−/−*^ or (B) *Krt14*^*Cre-*^*Sting*^*flox/flox*^ and *Krt14*^*Cre+*^*Sting*^*flox/flox*^ mice. Each dot represents an individual mouse. ^∗^ in the flow plots indicates significant difference compared to WT control mice. ^∗^p < 0.05, ^∗∗^p < 0.01, ^∗∗∗^p < 0.001, and ^∗∗∗∗^p < 0.0001 as calculated with two-way ANOVA with Holm-Šidák multiple comparison test. Data are represented as mean ± SEM. In (A), numbers correspond to the frequencies of gated populations ± SEM. Data are representative of two independent experiments using 4–6 mice per group. See also Figures S4 and S5.

**Figure S5:**
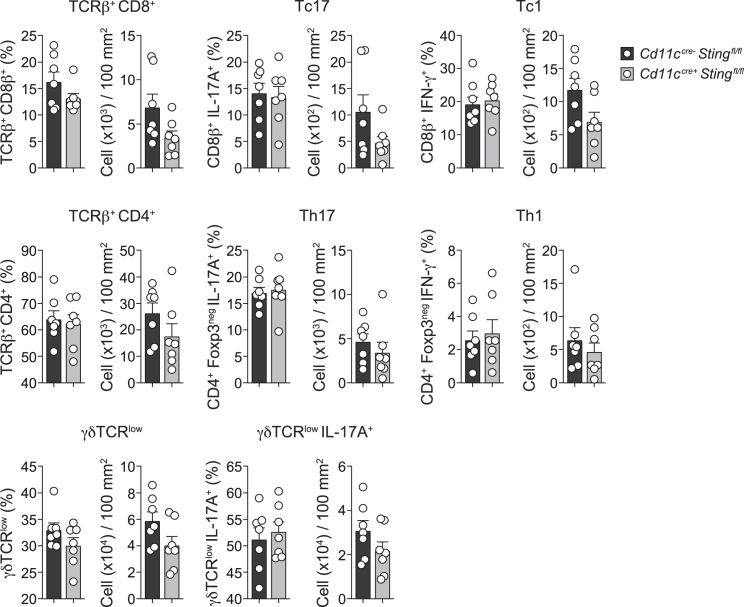
*S. epidermidis* induced T cells accumulation in the skin is independent of STING signaling in CD11c^+^ cells, related to Figure 4 Frequency and absolute number of CD8^+^ T cells, CD4^+^ T cells, γδTCR^low^ T cells, IL-17A^+^ (Tc17) or IFN-γ^+^ (Tc1) CD8^+^ T cells, IL-17A^+^ (Th17) or IFN-γ^+^ (Th1) CD4^+^ T cells and IL-17A^+^γδTCR^low^ cells in the ear pinnae of *Cd11c*^*cre-*^*Sting*^*flox/flox*^ and *Cd11c*^*cre-*^*Sting*^*flox/flox*^ mice two weeks post-association with *S. epidermidis*. Data are represented as mean ± SEM. Each dot represents an individual mouse.

We next sought to identify DNA sensors (Ma et al., 2018) potentially involved in these responses. Retroviruses have been previously shown to activate cGAS to produce cGAMP, which binds to and activates the adaptor protein STING (Gao et al., 2013). Under steady-state conditions, numbers of CD8^+^, CD4^+^ T cells, and IFN-γ-expressing CD8^+^ T cells (Tc1) were significantly decreased in the skin of *cGas*^*−*/−^ and *Sting*^*−*/−^ mice (Figure S4A). Thus, in SPF mice, the tonic accumulation of type 1 T cells in the skin depends on nucleic-acid-sensing pathways. Following *S. epidermidis* association, the absolute number of all T cell subsets analyzed as well as their ability to produce cytokines were significantly reduced in both *cGas*^*−*/−^ and *Sting*^*−*/−^ mice compared to WT controls (Figures 4A and S4B). The antiviral signature and upregulation of ERV expression by keratinocytes following *S. epidermidis* association supported a primary role for these cells in sensing ERV-derived cDNA molecules (Figures 1D, 2A, and 2B). Under steady-state conditions, deletion of STING in keratinocytes (*Krt14*^*Cre+*^*Sting*^*f/f*^) had no impact on microbial colonization or ERV expression (Figures S4C and S4D). On the other hand, lack of STING only in keratinocytes (but not in CD11c-expressing cells, which include dendritic cells [DCs]) was sufficient to reduce the numbers of Tc1 and Th1 cells within the skin (Figures 4B and S5). Deletion of STING in keratinocytes significantly reduced the number of all T cell subsets as well as cytokine production following association with *S. epidermidis* in a manner comparable to that seen in *Sting*^*−*/−^ mice and without impacting the level of ERV expression (Figures 4A, 4B, S4D, and S4E). Together, our results propose that keratinocyte responses to microbial colonization promotes a discrete upregulation of ERVs resulting in cGAS/STING activation that is required for the induction of homeostatic T cell responses to a skin microbe (Figure S4F).

### High-fat diet promotes enhanced ERV expression and inflammatory responses to the microbiota

**Figure 5:**
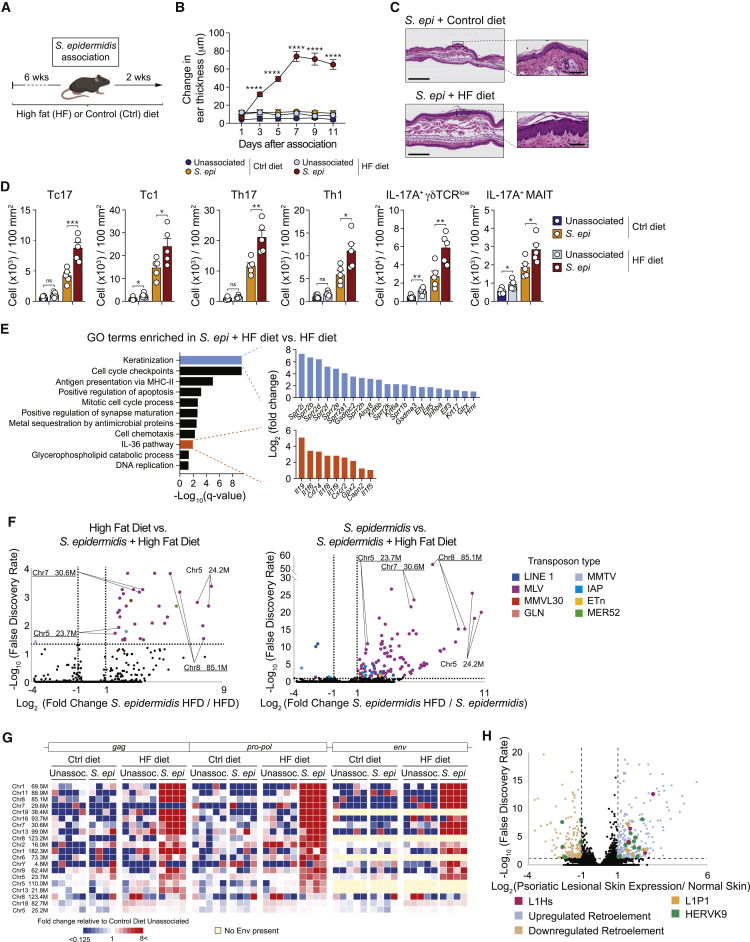
*S. epi* promotes skin inflammation and heightened retroelement expression in the context of high-fat diet (A) WT mice fed either a control (Ctrl) or a high-fat (HF) diet for 6 weeks were topically associated with *S. epi* or left unassociated. (B) Ear thickness measurement reported as the change in ear-skin thickness relative to baseline at day 0 (first day of *S. epi* association). (C) Representative image of hematoxylin-and-eosin staining of the skin from mice fed control diet or HF diet at day 14 after the first topical association with *S. epi*. Scale bars represent 300 μm or 50 μm (zoom in). (D) Absolute number of indicated lymphocyte subsets from ear pinnae of mice fed control diet or HF diet at day 14 after the first topical association. Each dot represents an individual mouse. (E) GO assignments of top 11 GO terms enriched in Sca-1^+^CD49f^+^ keratinocytes isolated from *S. epidermidis* associated versus unassoc. WT mice fed a HF diet at day 7 post-association. Upregulated genes related to GO terms keratinization (blue) and antigen presentation via MHC-II (orange) are shown. (F) Volcano plots of expressed retroelement loci from Sca-1^+^CD49f^+^ keratinocytes purified from the epidermis of unassoc. or *S. epi* associated WT mice fed a control or a HF diet, 7 days post-association. Underlined loci highlight retroelements with active reverse transcriptases. (G) RNA-seq expression levels of gene from differentially expressed ERV loci in sorted keratinocytes from unassoc. or *S. epi* associated mice fed control or HF diet at 7 days post-association. Multiple biological replicates are shown per condition. (H) Retroelement expression analyzed from previously published clinical cohort in which the transcriptome of normal skin was compared to psoriatic skin within the same patient. Retroelement families enriched in psoriatic lesions are specifically highlighted. ^∗^p < 0.05, ^∗∗^p< 0.01, ^∗∗∗^p < 0.001, and ^∗∗∗∗^p < 0.0001 as calculated with two-way ANOVA with Holm-Šidák multiple comparison test (B and D). Data are represented as mean ± SEM. Data are representative of two (C and E–G) or three (B and D) independent experiments using 4 to 5 mice per group or 54 paired samples (H) from 27 psoriasis patients. See also Figure S6.

**Figure S6:**
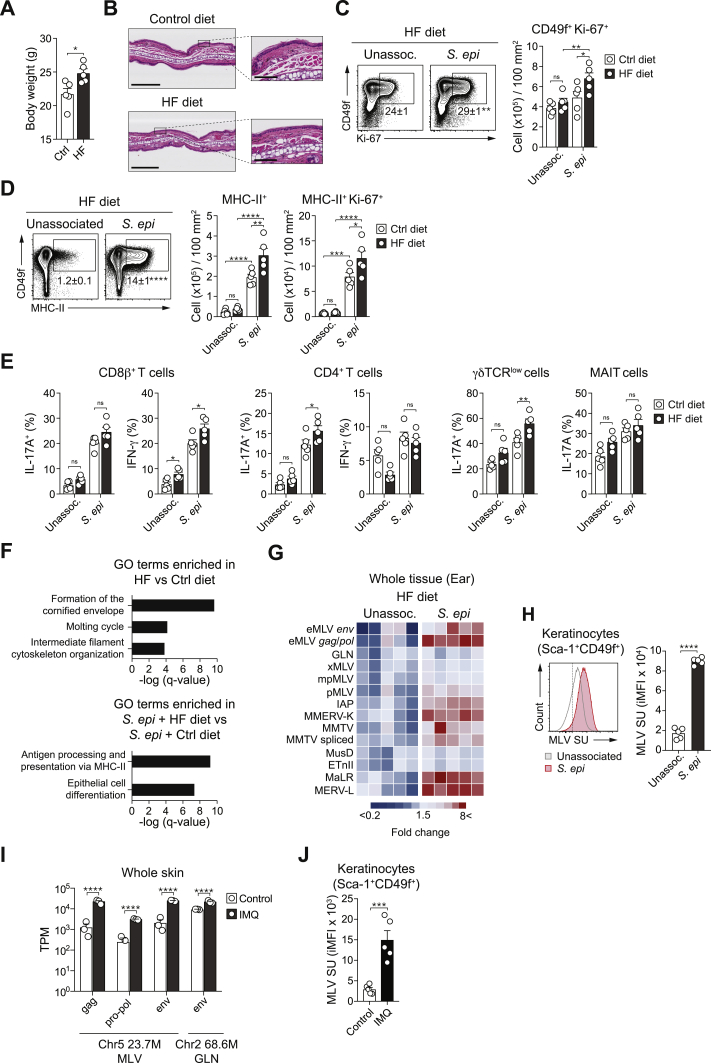
High-fat diet induces heightened ERVs expression and T cells responses following *S. epidermidis* colonization, related to Figure 5 WT mice were fed either a control (Ctrl) or a high-fat (HF) diet for 6 weeks and then topically associated with *S. epidermidis* (*S. epi*) or left unassociated. (A) Body weight measurement (g ± SEM) in mice after 6 weeks of diet regimen. (B) Representative image of hematoxylin-and-eosin staining of the ear pinnae from mice after 8 weeks of diet regimen. Scale bars = 300 μm or 50 μm (zoom in). (C) Frequency and absolute number of CD49f^+^Ki-67^+^ keratinocytes in the ear pinnae of unassociated (Unassoc.) and *S. epidermidis* (*S. epi*)-associated mice fed a control (Ctrl) or high-fat (HF) diet at day 14 post association. (D) Flow cytometry analysis of MHC-II expression and absolute number of CD49f^+^MHC-II^+^ and CD49f^+^MHC-II^+^Ki-67^+^ keratinocytes from unassociated (Unassoc.) or *S. epidermidis* (*S. epi*)-associated mice fed a control (Ctrl) or high-fat (HF) diet at 14 days post association. Plots in (C) and (D) were gated on live CD45^–^CD31^–^CD34^–^Sca-1^+^ cells. ^∗^ in the flow plot indicates significant difference between unassociated and *S. epi* group. (E) Frequency of IL-17A^+^ or IFN-γ^+^ CD8^+^ or CD4^+^ T cells and IL-17A^+^ γδTCR^low^ or MAIT cells in the ear pinnae from unassociated and *S. epidermidis*-associated mice fed a control (Ctrl) or a high-fat (HD) diet. All cells were stimulated with PMA and ionomycin. (F) Gene ontology assignments of top 3 or top 2 GO terms that were enriched in Sca-1^+^CD49f^+^ keratinocytes from WT mice fed a high-fat diet versus control diet and from *S. epidermidis*-associated (day 7) WT fed a high-fat diet versus control diet, respectively. (G) Heatmap showing transcript levels of the indicated ERVs measured by qRT-PCR of mRNA isolated from ear pinnae from unassociated or *S. epidermidis*-associated mice fed a high-fat (HF) diet at day 7 post association. Values were normalized to *Gapdh* expression in the same sample. (H) MLV SU expression detected by flow cytometry on the surface of Sca-1^+^CD49f^+^ keratinocytes from unassociated (Unassoc.) or *S. epidermidis*-associated mice fed a high-fat diet at day 7 post association. (I and J) WT mice were treated with imiquimod (IMQ) cream for 5 consecutive days or not (control). (I) Differential ERV levels analyzed by RNaseq in the ear pinnae 5 days after the beginning of IMQ treatment. Data was reanalyzed from our published study (Hurabielle et al., 2020). ^∗∗∗∗^ FDR ˂ 0.0001. (J) MLV SU expression detected by flow cytometry on the surface of Sca-1^+^CD49f^+^ keratinocytes from the ear pinnae 5 days after the beginning of IMQ treatment. ^∗^*P* < 0.05, ^∗∗^*P* < 0.01, ^∗∗∗^*P* < 0.001, ^∗∗∗∗^*P* < 0.0001 as calculated with Student’s t test (A), (H) and (J) or two-way ANOVA with Holm-Šidák multiple comparison test (C), (D) and (E). Data are represented as mean ± SEM. Each dot (A), C-E, H-J) represents an individual mouse. Data are representative of two (B), (F), (G), (H), (I) and (J) or three (A), (C), (D) and (E) independent experiments using 4-5 mice per group.

Although homeostatic immunity to the microbiota promotes immune fitness, dysregulated responses to these symbionts can also promote tissue inflammation (Belkaid and Hand, 2014). Heightened expression or sensing of ERVs has also been associated with inflammation and disease states (Stetson et al., 2008; Tokuyama et al., 2018). To test the possibility that ERVs could contribute to the pathogenic impact of the microbiota, we employed a model of dietary intervention, having previously shown that a high-fat diet can promote inflammatory response to the microbiota (Ridaura et al., 2018). The consumption of a diet rich in saturated fatty acids promotes inflammation in a nuclear factor κB (NF-κB)-dependent manner (Lionetti et al., 2014); a pathway previously linked to the induction of ERVs (Kassiotis and Stoye, 2016), and obesity increases the prevalence and severity of inflammatory disorders (Padhi and Garima, 2013). Mice were placed on a high-fat or control diet for 6 weeks prior to association with *S. epidermidis* (Figure 5A). At this time point, mice showed modest weight gain and did not develop skin inflammation (Figures S6A and S6B). High-fat diet alone did not impact keratinocyte proliferation or MHC-II expression (Figures S6C and S6D), whereas the number of Tc1, IL-17A-producing MAIT, and γδ T cells within the skin was increased compared to controls (Figure 5D). As previously described (Naik et al., 2015), *S. epidermidis* association of mice fed a regular diet did not result in inflammation (Figures 5B and 5C). On the other hand, when applied to mice fed a high-fat diet, *S. epidermidis* promoted a significant increase in skin thickening associated with a hyperplastic epidermis and hyperkeratosis (Figures 5B and 5C). Altered skin physiology was detectable as early as 3 days post-association and plateaued at 7 days and was associated with a significant increase in the number of all T cell subsets and cytokine production potential compared to controls (Figures 5B, 5D, and S6E).

In contrast to high-fat diet alone, *S. epidermidis* colonization under high-fat conditions increased the expression of genes associated with inflammatory responses in skin, including cell proliferation, positive regulation of apoptosis, chemotaxis, and the IL-36 signaling pathway (Figures 5E and S6F). Genes associated with keratinization and antigen presentation pathways were more significantly induced by *S. epidermidis* colonization under high-fat conditions than in the context of a regular diet (Figures 1D and 5E). Altered activation and inflammatory state of keratinocytes was further confirmed by a significant increase in MHC-II expression and proliferation in response to *S. epidermidis* in high-fat-diet-fed mice compared to controls (Figures S6C, S6D, and S6F).

We next assessed the level of ERV expression under the inflammatory conditions. Multiple families of ERV were strongly induced in the skin of mice fed a high-fat diet and colonized with *S. epidermidis* (Figure S6G). Further, mice colonized with *S. epidermidis* under a high-fat regimen highly expressed MLV ERV loci in keratinocytes relative to keratinocytes isolated from mice fed a high-fat diet alone (Figure 5F). The induced retroelements were distinct from those induced in the context of a high-fat diet or *S. epidermidis* alone (Figure 5F). Numerous ERV loci were induced by *S. epidermidis* colonization in the skin of mice fed a high-fat diet, all of which encoded a functional reverse transcriptase (Figure 5G). Consistent with these observations, keratinocytes from mice fed a high-fat diet and associated with *S. epidermidis* also expressed higher levels of MLV SU compared to mice fed a high-fat diet alone or mice fed a control diet and associated with *S. epidermidis* (Figures 2B and S6H). Thus, high-fat diet promotes heightened ERV expression in response to *S. epidermidis* association.

To assess a potential role for ERVs in other skin inflammatory settings, we evaluated ERV expression in an experimental model of psoriasis. Imiquimod treatment of mice (Hurabielle et al., 2020) was associated with a strong upregulation of the locus also upregulated in response to *S. epidermidis* (Chr5 23.7M), with imiquimod inducing expression of a defective locus (Chr2 68.6M) and heightened levels of MLV SU expression by keratinocytes compared to controls (Figures S6I and S6J). To assess the potential relevance of our findings to settings of human skin inflammation, we analyzed skin samples from psoriatic patients (Li et al., 2014). Our results revealed a strong induction of retroelement loci in psoriatic lesions relative to normal skin within the same patient and, more particularly, specific retroelement families, including two LINE-1 families, L1P1 and L1HS, and the ERV family HERVK9 (Figure 5H). Thus, skin inflammation in mice and humans can be associated with enhanced ERV expression.

### The cGAS/STING/IFN-I axis contributes to high-fat-diet-induced inflammatory responses to *S. epidermidis*

**Figure 6:**
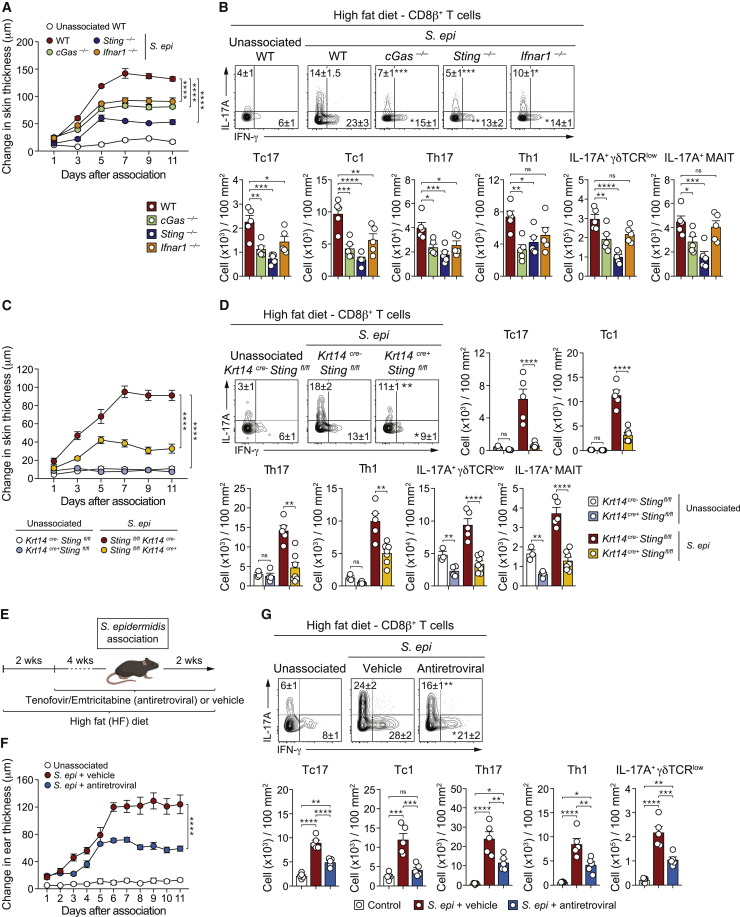
STING signaling and retroelement activity contribute to microbiota-induced inflammatory responses (A–D) WT, *cGas*^*−/−*^, *Sting*^*−/−*^, *Ifnar1*^*−/−*^, *Krt14*^*cre-*^*Sting*^*flox/flox*^, and *Krt14*^*cre+*^*Sting*^*flox/flox*^ mice fed a HF diet were topically associated with *S. epi* or left unassoc. (A and C) Ear thickness measurement reported as the change relative to baseline at day 0. (B and D) Frequency and absolute number of indicated lymphocyte subsets. (E) WT mice on HF diet were treated daily for 8 weeks with vehicle control (vehicle) or antiretrovirals starting at 2 weeks after the beginning of the HF-diet regimen. At 6 weeks post-HF diet, mice were associated or not with *S. epi*. (F) Ear thickness measurement. (G) Frequency of indicated lymphocyte subsets at 14 days post-association. Each dot represents an individual mouse. ^∗^ in the flow plots indicates significant difference compared to WT (B), *Krt14*^*Cre-*^*Sting*^*flox/flox*^ (D), and vehicle (G) *S. epi* associated conditions. ^∗^p < 0.05, ^∗∗^p < 0.01, ^∗∗∗^p < 0.001, and ^∗∗∗∗^p < 0.0001 as calculated with two-way ANOVA with Holm-Šidák multiple comparison test (A, C, and F) or one-way ANOVA with Holm-Šidák multiple comparison test (B, D, and G). Data are represented as mean ± SEM. Data are representative of two independent experiments using 3–7 mice per group. See also Figure S7.

**Figure S7:**
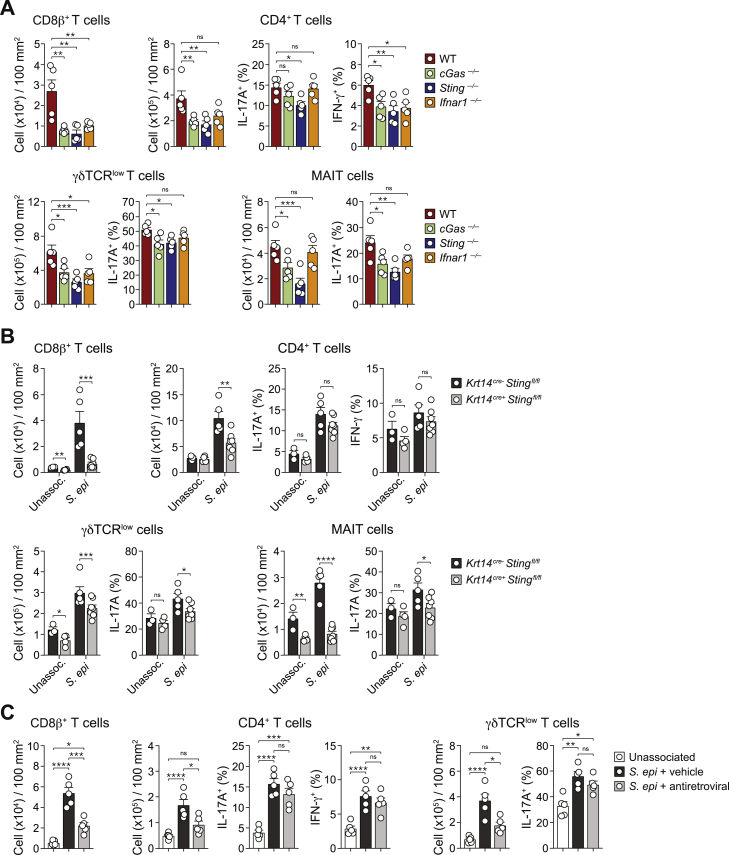
*S. epidermidis* induced aberrant skin inflammation in the context of high-fat diet is dependent on ERVs/cGas/Sting/IFN axis, related to Figure 6 (A-B) Absolute number of CD8^+^ T cells, CD4^+^ T cells, γδTCR^low^T cells and MAIT cells and frequency of IL-17A^+^ or IFN-γ^+^ CD4^+^ T cells and IL-17A^+^ γδTCR^low^ or MAIT cells in the ear pinnae from *S. epidermidis*-associated WT, *cGas*^*–/–*^, *Sting*^*–/–*^ and *Ifnar1*^*–/–*^ mice fed a high-fat diet (A) or from unassociated or *S. epidermidis*-associated *Krt14*^*cre-*^*Sting*^*flox/flox*^ and *Krt14*^*cre+*^*Sting*^*flox/flox*^ mice fed a high-fat diet (B). (C) WT mice fed a high-fat diet (HF) were treated daily with either vehicle control (vehicle) or a combination of tenofovir disoproxil fumarate and emtricitabine (antiretroviral), beginning at 2 weeks post HF diet for a total of 8 weeks. At 6 weeks post HF diet, mice were topically associated with *S. epidermidis* (*S. epi*) or left unassociated. Absolute number of CD8^+^ T cells, CD4^+^ T cells and γδTCR^low^ T cells and frequencies of IL-17A^+^ or IFN-γ^+^ CD4^+^ T cells and IL-17A^+^ γδTCR^low^ cells. All cells were stimulated with PMA and ionomycin. Data are represented as mean ± SEM. Each dot represents an individual mouse. ^∗^*P* ˂ 0.05, ^∗∗^*P* < 0.01, ^∗∗∗^*P* < 0.001, ^∗∗∗∗^*P* < 0.0001 as calculated with one-way ANOVA (A) and (C) or two-way ANOVA with Holm-Šidák multiple comparison test (B). Data are representative of two independent experiments using 3-7 mice per group.

We next assessed whether, in the context of high-fat diet, IFN-I and cytosolic nucleic acid sensors contributed to the pathology induced by *S. epidermidis*. The ability of *S. epidermidis* to cause skin inflammation and promote T cell responses in the context of a high-fat diet was significantly reduced in *cGas*^*−/−*^, *Sting*^*−/−*^, and *Ifnar1*^*−/−*^ mice (Figures 6A, 6B, and S7A). Defects in DNA sensing more significantly impacted T cell responses to *S. epidermidis* than defects in IFN-I signaling, supporting the idea that other inflammatory mediators contributed to skin inflammation under these conditions (Figures 6B and S7A).

We next assessed the contribution of keratinocyte-specific nucleic acid sensing. Skin pathology induced by *S. epidermidis* in mice fed a high-fat diet was significantly reduced in *Krt14*^*Cre+*^*Sting*^*fl/fl*^ as compared to control *Krt14*^*Cre-*^*Sting*^*fl/fl*^ mice (Figure 6C). In mice fed a high-fat diet, the absolute number of IL-17A-producing γδ T cells and MAIT cells, but not other cell subsets, was significantly reduced in *Krt14*^*Cre+*^*Sting*^*fl/fl*^ compared to controls (Figure 6D). Following *S. epidermidis* association, the absolute number of all T cell subsets induced by *S. epidermidis* was significantly reduced in the skin of high-fat fed *Krt14*^*Cre+*^*Sting*^*fl/fl*^ mice compared to controls (Figure 6D). The ability of CD8^+^ T cells to produce IL-17A and IFN-γ and MAIT and γδ T cells to produce IL-17A was also significantly reduced in *Krt14*^*Cre+*^*Sting*^*fl/fl*^ mice compared to control mice (Figures 6D and S7B). Thus, DNA sensing by keratinocytes contributes to the ability of the microbiota to promote inflammation in the context of metabolic alteration.

### Reverse transcription promotes high-fat-diet-induced inflammatory responses to *S. epidermidis*

To evaluate a potential role for ERVs and reverse transcriptase in driving the inflammatory responses caused by microbes under high-fat-diet conditions, mice were treated with vehicle or antiretrovirals prior to *S. epidermidis* association and thereafter (Figure 6E). Under these settings, skin inflammation caused by *S. epidermidis* was significantly reduced compared to controls (Figure 6F). This level of reduction was comparable to that observed in STING-deficient mice or that with a keratinocyte-specific STING deletion (Figures 6A and 6C). Antiretroviral treatment was also associated with significant reduction in the number of all T cell subsets as well as a decrease in the ability of CD8^+^ T cells to produce cytokines (Figures 6G and S7C). Taken together, these data support the idea that the ERV/cGAS/STING/IFN-I axis plays a fundamental role in context-specific responses to the skin microbiota.

## Discussion

Here, we propose that the host endogenous virome dominantly controls the alliance between the microbiota and the immune system. Our results also propose that the level of ERV expression fundamentally controls the threshold of tissue activation and dictates the ability of the host to respond in homeostatic or inflammatory manner to the microbiota.

The mechanisms underlying the physiological initiation of homeostatic immunity to the microbiota are unclear. Our present work supports the idea that the host endogenous virome may represent a fundamental adjuvant of these responses. Thus far, our model proposes that discrete sensing of the microbiota by keratinocytes promotes the expression of defined ERVs and that these ERVs reverse transcribe, resulting in cytosolic accumulation of ERV-derived cDNA. The cGAS/STING signaling pathway then senses these cDNAs. These discrete keratinocyte hot spots could promote a favorable environment for DCs to capture microbiota-derived antigens and migrate to lymph nodes. Following priming, commensal-specific T cells migrate back to the skin, where their accumulation and ERV-activated keratinocytes may also promote T cell function. In the context of non-classical T cells that expand in tissues, such as MAIT cells (Constantinides et al., 2019), local responses to ERVs may be sufficient to control their accumulation in the skin, post-microbiota association. The result of this multi-kingdom dialog is the induction and accumulation of a network of commensal-specific T cells able to broadly promote tissue physiology, including tissue repair (Figure S4F).

Although ERVs represent a sizable proportion of the mammalian genome, most ERVs are inactive (Johnson, 2015; Kassiotis and Stoye, 2016). However, many ERVs have retained retroviral characteristics, including the ability to reverse transcribe and, in some cases, form virus-like particles (Kassiotis and Stoye, 2016). As such, these elements are under constant immune pressure and in a state of equilibrium with the host, a dialog proposed to contribute to the tonic activation of the immune system (Hu et al., 2015; Stetson et al., 2008; Young et al., 2012a). TLR signaling induces these elements (Young et al., 2014), a phenomenon that we confirmed also occurs in keratinocytes. In line with our observations, *S. epidermidis* derived lipoproteins and teichoic acids, together with host expression of TLR2/Dectin-1, are required for the optimal induction of commensal-specific T cells (Chen et al., 2019). In agreement with a role for these pathways in promoting ERV expression, host expression of TLR2 and *S. epidermidis* expression of teichoic acids and lipoproteins are required for optimal induction of ERVs in the skin. Of particular interest, *S. epidermidis* colonization promoted the selective induction of ERVs. We speculate that these defined sequences may have lost a repressor sequence or gained a transcription factor binding site that is active in keratinocytes. In support of this hypothesis, the strong responsiveness of Chr5, 23.7M to LPS stimulation in lymphocytes is, at least in part, due to the LPS responsiveness of the lncRNA AI506816 promoter near the Chr5, 23.7M integration site (Panova et al., 2020; Young et al., 2012a). Of interest, the induction of ERV families was variable among skin commensals, a phenomenon that could contribute to the ability of defined microbes to impose unique immune signatures. These observations also raise an intriguing possibility that microbe- and cell-type-specific expression of ERVs may control local immune microenvironments. In support of this, expression of retroelements is tissue specific and expression of defined ERV families is lost in the absence of the microbiota (Young et al., 2012a, 2014). Whether a role for ERVs is restricted to sites with low microbial biomass, such as the skin or the lung remains unclear, but based on the prevalence of retroelements in the mammalian genomes, all responses to the microbiota may be, at least in part, ERV dependent.

Antiretroviral treatment not only reduced T cell accumulation within the skin but also impaired subsequent responses of keratinocytes, including decreased expression of genes involved in antiviral responses and wound healing. In agreement, antiretroviral treatment had a significant impact on *S. epidermidis* accelerated wound repair. Epithelial cells not only dictate the induction of responses directed toward the microbiota but also provide specific checkpoints allowing functional licensing and retention of T cells within tissues (Naik et al., 2012; Sano et al., 2015; Tanoue et al., 2019). For instance, we previously showed that MHC-II expression by keratinocytes was required for homeostatic Th1 responses to the microbiota (Tamoutounour et al., 2019). Indeed, the microbiota promoted the expression of genes associated with T cell homing, as well as numerous genes associated with MHC class I and MHC-II antigen presentation and processing pathways, with a substantial fraction of these impacted by antiretroviral treatment. Of note, MHC-II^+^ keratinocytes express elevated levels of MLV SU, indicating that these cells may represent hot spots for the initiation (e.g., via DC activation) and/or accumulation (e.g., via chemokine production) of T cell responses to the microbiota. Further, ERV-activated keratinocytes may play an important role in the local presentation of microbiota-derived antigens to commensal-specific T cells.

In agreement with a role for STING, IFN-I also promoted commensal-specific T cell responses. Within the skin, IFN-I could act on numerous cell types, including keratinocytes, DCs, or T cells (Lazear et al., 2019). The microbiota has been shown to promote a broad antiviral program within tissues, which was associated with their ability to enhance protective immunity against viral infections (Abt et al., 2012; Di Domizio et al., 2020; Gutierrez-Merino et al., 2020; Schaupp et al., 2020). The skin microbiota also induces IFN-I to promote tissue repair (Di Domizio et al., 2020). Within the gut, microbiota-induced IFN-I has also been proposed to rely on viral sensors, such as STING and mitochondrial antiviral signaling protein (MAVS) (Gutierrez-Merino et al., 2020). Thus, the ability of the microbiota to promote IFN-I-dependent responses at all barrier sites, including protective immunity and tissue repair, may be controlled by ERV activity and innate responses against these endogenous retroelements.

Pathologies that increasingly affect humans, such as allergies, autoimmunity, and inflammatory disorders, have all been linked to altered composition or responses to the microbiota (Blander et al., 2017). Although a pathogenic link between ERVs and the microbiota has not been previously reported, retroelements are well recognized for their pathogenic potential (Attig et al., 2017). Our results propose that enhanced ERV expression may contribute to inflammatory responses to the microbiota. Notably, we showed that antiretroviral treatment or specific deletion of STING in keratinocytes alleviated inflammation caused by *S. epidermidis* in mice fed a high-fat diet. Our data from murine model of psoriasis and human psoriatic lesions also support the idea that skin inflammation may be generally associated with enhanced expression of ERVs. These results are in line with previous work demonstrating that ERV-derived nucleic acids and protein expression are frequently increased in numerous settings, including infection, autoimmunity, and cancer (Stetson et al., 2008; Tokuyama et al., 2018). Furthermore, increased retroelement expression coupled with mutations in cytosolic-DNA-sensing genes have been implicated in inflammation and autoimmunity (Crowl et al., 2017). Aberrant accumulation of self-DNA promotes inflammatory processes in a cGAS-STING-dependent manner (Gao et al., 2013; Pokatayev et al., 2016; Stetson et al., 2008), and a link between ERVs and cGAS has been demonstrated in the context of defined inflammatory mouse models (Gao et al., 2015) and proposed in defined human settings (Mustelin and Ukadike, 2020).

Whether increased levels of ERV expression above a defined set point could be sufficient to promote inflammation remains to be addressed, but tissue context is a likely critical determinant. Indeed, altered activation status of keratinocytes imposed by nutritional stress or altered regulatory landscape is expected to also impact responses to ERVs. How a high-fat diet primes the skin for enhanced ERVs expression in the context of microbial exposure remains unclear. Numerous factors associated with metabolic alterations, including enhanced cytokine levels (e.g., IL-1), enhanced microbial translocation, or responses to saturated fatty acids (Reilly and Saltiel, 2017), could contribute to an altered tissue state of activation. Although inflammation caused by *S. epidermidis* in mice fed a high-fat diet was significantly reduced following antiretroviral treatment or in mice lacking STING specifically in keratinocytes, this reduction was not absolute, supporting the idea that additional cellular partners and innate pathways could also contribute to these inflammatory processes. Of relevance to our findings, obesity has been linked to both altered microbiota composition and increased severity and development of numerous inflammatory skin disorders, including atopic dermatitis and psoriasis (Padhi and Garima, 2013; Zhang et al., 2015). How microbiota-induced ERV expression contributes to the etiology of these disorders remains to be addressed.

Together, our work proposes that tissue physiology may be controlled by a fundamental alliance between the exogenous microbiota and the endogenous virome. Our work also supports the idea that ERV responses to the microbiota should be integrated into our understanding of diseases and inflammatory states.

### Limitations of study

In our study, we showed that endogenous retroviral transcripts were induced by various skin commensals. Whether these specific ERV loci are the primary molecules being sensed by cGAS/STING needs to be further confirmed. It is possible that numerous retroelements are tonically sensed by cGAS/STING and that antiretroviral treatment reduces the sensing of all elements. As ERV RNA is also sensed by immune cells (via melanoma differentiation-associated protein 5 [MDA5]), we cannot exclude a possible role for sensing of ERV RNA in commensal induced T cell immunity. Additionally, we present data showing that retroelements are induced in lesional keratinocytes from patients with the autoinflammatory disorder psoriasis. However, at this time, it is unclear whether retroelement sensing plays a role in the pathogenesis of this disease.

## STAR★Methods

### Key resources table

**Table:** 

REAGENT or RESOURCE	SOURCE	IDENTIFIER
**Antibodies**		

Anti-mouse CD3ε (145-2C11), BV605	Biolegend	Cat #100351; RRID: AB_2565842
Anti-mouse CD4 (RM4-5), AF700	Thermo Fisher Scientific (eBioscience)	Cat #56-0042-82; RRID: AB_494000
Anti-mouse CD4 (RM4-5), BV510	BioLegend	Cat #100559; RRID: AB_2562608
Anti-mouse CD8α, PE (53-6.7)	Thermo Fisher Scientific (eBioscience)	Cat# 12-0081-83; RRID: AB_465531
Anti-mouse CD8β (eBioH35-17.2), PerCP-eFluor710	Thermo Fisher Scientific (eBioscience)	Cat #46-0083-82; RRID: AB_10669709
Anti-mouse CD8β (H35-17.2), BV605	BD Biosciences	Cat #740387; RRID: AB_2740117
Anti-mouse CD16/32 (clone 2.4G2)	Bio-X-Cell	Cat #BE0307; RRID: AB_2736987
Anti-mouse CD31 (MEC13.3), PerCP-Cy5.5	Biolegend	Cat #102522; RRID: AB_2566761
Anti-mouse CD34 (RAM34), eFluor 660	Thermo Fisher Scientific (eBioscience)	Cat #50-0341-82; RRID: AB_10596826
Anti-mouse CD45 (30-F11), APC-eFluor 780	Thermo Fisher Scientific (eBioscience)	Cat #47-0451-82; RRID: AB_1548781
Anti-mouse CD45 (30-F11), BV510	Biolegend	Cat #103138; RRID: AB_2563061
Anti-mouse CD49f (eBioGoH3), PE	BD Biosciences	Cat #313612; RRID: AB_893373
Anti-mouse CD90.2 (53-2.1), BV605	BioLegend	Cat #140318; RRID: AB_2650924
Anti-mouse CD90.2 (30-H12), BV785	BioLegend	Cat #105331; RRID: AB 2562900
Anti-mouse FOXP3 (FJK-16 s), FITC	Thermo Fisher Scientific (eBioscience)	Cat #11-5773-82; RRID: AB_465243
Anti-mouse IFN-γ (XMG1.2), eFluor 450	Thermo Fisher Scientific (eBioscience)	Cat #48-7311-82; RRID: AB_1834366
Anti-mouse IFNAR-1 (MAR1-5A3)	Bio-X-Cell	Cat #BE0241; RRID: AB_2687723
Anti-mouse IL-17A (TC11-18H10.1), PE-Cy7	Biolegend	Cat #506922; RRID:AB_2125010
Anti-mouse Ki-67 (SolA15), eFluor 450	Thermo Fisher Scientific (eBioscience)	Cat #48-5698-82; RRID: AB_11149124
Anti-mouse MHC-II (M5/114.15.2), AF700	Thermo Fisher Scientific (eBioscience)	Cat #56-5321-82; RRID: AB_494009
Anti-mouse Sca-1 (D7), FITC	Biolegend	Cat #108106; RRID: AB_313343
Anti-mouse TCRβ (H57-597), BUV737	BD Biosciences	Cat #612821; RRID:AB_2870145
Anti-mouse TCRγδ (eBioGL3), PE-CF594	BD Biosciences	Cat #563532; RRID: AB_2661844
Biotin Mouse Anti-Rat IgG2a (RG7/1.30)	BD Biosciences	Cat #553894; RRID: AB_395122
CD49f (Integrin alpha 6) Monoclonal Antibody (eBioGoH3 (GoH3)	Thermo Fisher Scientific (eBioscience)	Cat #14-0495-82; RRID: AB_891480
Goat Anti-Chicken IgG	Jackson ImmunoResearch Laboratories	Cat #103-605-155; RRID:AB_2337392
Keratin 14 Polyclonal Chicken Antibody, Purified	Biolegend	Cat #906001; RRID: AB_2565055
Mouse IgG1 isotype control (MOPC-21)	Bio-X-Cell	Cat #BP0083; RRID: AB_1107784
Normal Goat Serum	Jackson ImmunoResearch Laboratories	Cat #005-000-121; RRID: AB_2336990
Rat Gamma Globulin	Jackson ImmunoResearch Laboratories	Cat #012-000-002; RRID: AB_2337135

**Bacterial strains**		

*Staphylococcus aureus* AD04.E17	Laboratory of Dr. Julie Segre (NHGRI/NIH)	N/A
*Staphylococcus epidermidis* NIHLM087	Laboratory of Dr. Julie Segre (NHGRI/NIH)	N/A
*Staphylococcus epidermidis* NIHLM087 Δ*tagO*	Laboratory of M.A.F.	N/A
*Staphylococcus epidermidis* NIHLM087 Δ*lgt*	Laboratory of M.A.F.	N/A
*Staphylococcus xylosus* 42C08	Laboratory of Dr. Julie Segre (NHGRI/NIH)	N/A

**Chemicals, peptides, and recombinant proteins**		

2-Mercaptoethanol (55 mM)	Thermo Fisher Scientific	Cat #21985923
2-Mercaptoethanol	Sigma-Aldrich	Cat #M6250
5-OP-RU mMR1-streptavidin-phycoerythrin	NIH Tetramer Core Facility	N/A
BSA	Sigma-Aldrich	Cat #A3059-500G
Brefeldin A (GolgiPlug)	BD Biosciences	Cat #555029; RRID: AB_2869014
Chelex 100 Chelating Resin	Bio-Rad	Cat #142-2842
Columbia Blood Agar with 5% Sheep Blood	Thermo Fisher Scientific	Cat #R01215
DAPI	Sigma-Aldrich	Cat #D9542
Dimethyl sulfoxide (DMSO)	Sigma	Cat #D2650
Dispase II	Thermo Fisher	Cat #17105-041
DNase I	Sigma-Aldrich	Cat #DN25-5G
EDTA (0.5M)	Corning	Cat #46-034-CI
Emtricitabine	Fisher Scientific	Cat #AC462070050
f-MIIINA peptide	Genscript	N/A
f-MIIINA:H2:M3-streptavidin-phycoerythrin	NIH Tetramer Core Facility	N/A
Fetal Bovine Serum (FBS)	GE Healthcare Hyclone	Cat #SH30070.03
Fetal Bovine Serum (FBS)	Seradigm Life Sciences	Cat #89510-186
Formalin	Sigma-Aldrich	Cat #HT501128
HBSS without Calcium and Maganesium	Corning	Cat #21-022-CV
HEPES	Corning	Cat #25-060-Cl
Imiquimod (Aldara Cream 5%)	3M Health Care N/A	N/A
Ionomycin	Sigma-Aldrich	Cat #I0634-5MG
L-Glutamine	Corning	Cat #25-005-Cl
Liberase TL	Sigma-Aldrich	Cat #05401020001
MEM Non-essential Amino Acids (100X)	Corning	Cat #25-025-CI
O.C.T. Embedding Medium	Fisher Scientific	Cat #23-730-571
Paraformaldehyde	Electron Microscopy Sciences	Cat #15714-S
PBS without Calcium and Magnesium	Corning	Cat #21-040-CM
Pennicillin-Streptomycin (100X)	Corning	Cat #30-002-Cl
Phorbol 12-myristate 13-acetate (PMA)	Sigma-Aldrich	Cat #P8139-10MG
ProLong Gold Antifade Mountant	Molecular Probes	Cat #P36930
Purified LTA from *S. aureus*	Invivogen	Cat #tlrl-pslta
R848 (Resiquimod)	Invivogen	Cat #tlrl-r848-5
RNAlater	Sigma-Aldrich	Cat #R0901-100ML
RPMI 1640 medium with L-Glutamine	Corning	Cat #10-040-CV
Sodium Pyruvate (100X)	Corning	Cat #25-000-Cl
Streptavidin PE/Cyanine7	Biolegend	Cat #405206;
Tenofovir Disoproxil Fumarate	Fisher Scientific	Cat #AC461250250
Triton X	Sigma-Aldrich	Cat #T9284
TRIzol reagent	Thermo Fisher Scientific	Cat #15596018
Trypsin-EDTA (0.25%), phenol red	Thermo Fisher Scientific	Cat #25200056
Tryptic Soy Broth	KD Medical	Cat #CUS-0279
Ultrapure LPS from *E. coli* O111:B4	Invivogen	Cat #tlrl-3pelps

**Critical commercial assays**		

BD Cytofix/Cytoperm	BD Biosciences	Cat #554722
BD Perm/Wash	BD Biosciences	Cat #554723
Chromium i7 Multiplex Kit	10X Genomics	Cat #PN-120262
Chromium Single Cell A Chip Kit	10X Genomics	Cat #PN-120236
Chromium Single Cell 3′ Library & Gel Bead Kit v2	10X Genomics	Cat #PN-120237
Foxp3 / Transcription Factor Staining Buffer Set	Thermo Fisher Scientifc (eBiocience)	Cat #00-5523-00
HiSeq 3000/4000 SBS Kit	Illumina	Cat #FC-410-1002
LIVE/DEAD Fixable Blue Dead Cell Stain Kit	Thermo Fisher Scientific	Cat #L23105
NextSeq 500/550 High Output Kit v2 (75 cycles)	Illumina	Cat #20024906
Omniscript RT Kit	QIAGEN	Cat #205111
RNeasy FibrousTissue Mini Kit	QIAGEN	Cat #74704
RNeasy Plus Micro Kit	QIAGEN	Cat #74034
TruSeq Stranded Total RNA Library Prep Human/Mouse/Rat (96 samples)	Illumina	Cat #20020597

**Deposited data**		

Raw RNA-seq data	This paper	GEO: GSE160688
Psoriasis Raw RNA-seq data	Li et al., 2014	GEO: GSE54456

**Experimental models: Organisms/strains**		

Mouse: C57BL/6J	Jackson Laboratory	Mouse Strain: Jax 000664
Mouse: B6(C)-*Cgas*^*tm1d(EUCOMM)Hmgu*^/J	Jackson Laboratory	Mouse Strain: Jax 026554
Mouse: B6.129S2-*Ifnar1*^*tm1Agt*^/Mmjax	Jackson Laboratory	Mouse Strain: Jax 32045
Mouse: B6.Cg-Tg(Itgax-cre)1-1Reiz/J	Jackson Laboratory	Mouse Strain: Jax 008068
Mouse: B6N.Cg-Tg(KRT14-cre)1Amc/J	Jackson Laboratory	Mouse Strain: Jax 018964
Mouse: B6;SJL-*Sting1*^*tm1.1Camb*^/J	Jackson Laboratory	Mouse Strain: Jax 025805
Mouse: B6.129-*Tlr2*^*tm1Kir*^/J	Jackson Laboratory	Mouse Strain: Jax 004650

**Oligonucleotides**		

Primers for eMLV *env*, see Table S1	Young et al., 2012b	N/A
Primers for eMLV *gag/pol*, see Table S1	Young et al., 2012a	N/A
Primers for ETnII, see Table S1	Karimi et al., 2011	N/A
Primer: *Gapdh* Forward AGGC TCAAGGGCTTTTAAGG	This paper	N/A
Primer: *Gapdh* Reverse ATCC TGTAGGCCAGGTGATG	This paper	N/A
Primers for GLN, see Table S1	Karimi et al., 2011	N/A
Primers for IAP, see Table S1	Collins et al., 2015	N/A
Primers for MaLR, see Table S1	Karimi et al., 2011	N/A
Primers for Mervl Pol, see Table S1	Macfarlan et al., 2011	N/A
Primers for MMERVK. See Table S1	Stoltz et al., 2019	N/A
Primers for MMTV, see Table S1	Yin et al., 2011	N/A
Primers for MMTV spliced, see Table S1	Young et al., 2012a	N/A
Primers for mpMLV. See Table S1	Yoshinobu et al., 2009	N/A
Primers for MusD, See Table S1	Karimi et al., 2011	N/A
Primers for pMLV. See Table S1	Yoshinobu et al., 2009	N/A
Primers for xMLV, see Table S1	Yoshinobu et al., 2009	N/A

**Software and algorithms**		

Cell Ranger software version 4.0.1	10X Genomics	RRID: SCR_017344
DESeq2 package version 2.3.11	https://bioconductor.org/packages/release/bioc/html/DESeq2.html	RRID: SCR_015687
FastQC software package version 0.11.5	Babraham Bioinformatics	RRID: SCR_014583
Fiji image processing package	Schindelin et al., 2012	RRID: SCR_003070
FlowJo software version 10.6.1	Treestar	RRID: SCR_008520
HOMER software version 4.11	http://homer.ucsd.edu/	RRID: SCR_010881
Imaris software version	Bitplane	RRID: SCR_007370
Metascape	http://metascape.org/gp/index.html#/main/step1	RRID: SCR_016620
Prism software version 9	GraphPad	RRID: SCR_002798
R version 4.05	http://www.r-project.org	N/A
Seurat package version 4.0	Hao et al., 2021	RRID: SCR_007322
STAR aligner version 2.7.5	Dobin et al., 2013	RRID: SCR_015899

**Other**		

Adjusted Calories Diet (60% Fat Kcal, Irradiated) - High Fat Diet	Envigo Teklad Diets	Cat #TD.06414
Control Diet (10% Fat Kcal, Irradiated)	Envigo Teklad Diets	Cat #TD.150064

### Resource availability

#### Lead contact

Further information and requests for resources and reagents should be directed to and will be fulfilled by the Lead Contact, Yasmine Belkaid (ybelkaid@niaid.nih.gov).

#### Materials availability

The *S. epidermidis* NIHLM087 *ΔtagO* and NIHLM087 *Δlgt* mutants used in this study are available upon request.

#### Data and code availability

RNA Sequencing datasets generated during this study are available at the NCBI GEO: GSE160688. The published data are available at NCBI GEO under the accession GSE54456.

### Experimental model and subject details

#### Mice

C57BL/6 J (wild-type [WT] mice), *cGas*^*–/–*^ (B6(C)-*Cgas*^tm1d(EUCOMM)Hmgu^/J), *Sting*^*–/–*^ (B6(Cg)-Sting1^tm1.2Camb^/J), *Krt14*^*Cre+*^ (B6N.Cg-Tg(KRT14-cre)^1Amc^/J), *Cd11c*^*Cre+*^ [B6.Cg-Tg(Itgax-cre)1-1Reiz/J], *Tlr2*^*–/–*^ (B6.129-Tlr2^tm1Kir^/J) and *Sting*^*flox/flox*^ *(*B6;SJL-*Sting1*^tm1.1Camb^/J) mice were purchased from the Jackson Laboratory. *Ifnar1*^*–/–*^ (B6.129S2-*Ifnar1*^*tm1Agt*^/Mmjax) were obtained either through the NIAID-Taconic exchange program or from Jackson Laboratory. For experiments with knockout mice, littermate controls were used as wild-type controls, and/or wild-type (C57BL/6J) and knockout mice were co-housed at 3-5 weeks of age for 2-3 weeks in the same cage, prior to the start of experimental manipulations. All mice were bred and maintained under SPF conditions at an American Association for the Accreditation of Laboratory Animal Care (AAALAC)–accredited animal facility at NIAID and housed in accordance with the procedures outlined in the Guide for the Care and Use of Laboratory Animals. All experiments were performed at NIAID under an Animal Study Proposal (LHIM3E) approved by the NIAID Animal Care and Use Committee. Sex- and age-matched mice between 4 and 8 weeks of age were used for each experiment. Female and male mice were used for experiments involving conditional knockout mice, while only female mice were used for experiments involving only conventional mice.

#### S. epidermidis culture and topical association of mice. Staphylococcus epidermidis

NIHLM087 (wt), *S. epidermidis* NIHLM087 *ΔtagO* (Chen et al., 2019), *S. epidermidis* NIHLM087 *Δlgt* (Chen et al., 2019), *Staphylococcus aureus* AD04.E17 (Byrd et al., 2017) and *Staphylococcus xylosus* 42C08 (Linehan et al., 2018) were cultured for 18 h in Tryptic Soy Broth at 37°C without shaking. For topical association of bacteria, each mouse was associated with a bacterial suspension (∼10^9^ colony-forming unit [CFU]/mL) across the surface of the ear pinnae or across the entire back skin using a sterile cotton swab. Application of bacterial suspension was repeated 4 times every other day for a total of four times or performed daily for 7 days as indicated before analysis.

### Method details

#### *In vivo* treatment with blocking antibodies

Mice were injected intraperitoneally with 1 mg of either anti-mouse IFNAR1 antibody (clone MAR1-5A3; BioXCell) or mouse IgG1 isotype control (clone MOPC-21; BioXCell) 1 day before the initial application of *S. epidermidis* NIHLM087; each mouse then received 0.5 mg of either antibody at days 3, 6, 9 and 12 post association.

#### Anti-retroviral treatment of mice

Mice were provided a combination of Tenofovir disproxil fumarate and Emtricitabine (both from ACROS Organics) based on the effective doses previously reported in the literature or human-to-animal dose translation studies (100 mg/kg for Tenofovir and 60 mg/kg Emtricitabine; Choudhary et al., 2009; Denton et al., 2012; Reagan-Shaw et al., 2008). Both antiretroviral drugs were dissolved in water and a cocktail containing 12.5 mg/mL of Tenofovir and 7.5 mg/mL of Emtricitabine was administrated daily by gavage to mice in a total volume 200 μL starting 7 days prior to *S. epidermidis* colonization or 2 weeks after the start of the high-fat diet regimen.

#### Anti-retroviral treatment of S. epidermidis

Tenofovir disproxil fumarate and Emtricitabine (both from ACROS Organics) were dissolved in Tryptic Soy Broth, and then mixed at concentrations ranging from 10 mg/ml to 0.64 μg/ml with a culture of *S. epidermidis* NIHLM087 at an OD600 of 0.1. The bacterial culture was then incubated for 12 hours and the OD600 was measured every 10 minutes using a Biotek Synergy HTX plate reader.

#### Back-skin wounding and epifluorescence microscopy of wound tissue

Wounding and quantitation of wound healing were performed as previously described (Keyes et al., 2016). Briefly, male mice in the telogen phase of the hair cycle were anesthetized with ketamine/xylazine, shaved with clippers and punch biopsies were performed on the back skin. A 6-mm biopsy punch was used to partially perforate the skin and iris scissors were then used to cut epidermal and dermal tissue to create a full thickness wound in a circular shape. Back-skin tissue was excised 5 days after wounding, fixed in 4% paraformaldehyde in PBS, incubated overnight in 30% sucrose in PBS, embedded in OCT compound (Tissue-Tek), frozen on dry ice, and cryo-sectioned (20-mm section thickness). Sections were fixed in 4% paraformaldehyde in PBS, rinsed with PBS, permeabilized with 0.1% Triton X-100 in PBS (Sigma-Aldrich), and blocked for 1 hour in blocking buffer (2.5% Normal Goat Serum, 1% BSA, 0.3% Triton X-100 in PBS). Sections were then incubated overnight with the primary antibody chicken anti-mouse Keratin 14 antibody (Poly9060, BioLegend) diluted 1:400 in blocking buffer and in presence of rat gamma globulin and anti-CD16/32. After washing with PBS, a secondary antibody conjugated with Alexa647 (goat anti-chicken, Jackson ImmunoResearch) was added for 1 hour at room temperature. Slides were then washed with PBS, counterstained with DAPI and mounted in Prolong Gold. Wound images were captured with a Leica DMI 6000 widefield epifluorescence microscope equipped with a Leica DFC360X monochrome camera. Tiled and stitched images of wounds were collected using a 20 × /0.4NA dry objective. Images were analyzed using Imaris software (Bitplane).

#### Bacteria quantitation

The ear skin of topically associated or unassociated control mice were swabbed with a sterile cotton swab previously soaked in tryptic soy broth. Swabs were streaked on Columbia blood agar plates. Plates were then placed at 37°C under aerobic conditions for 18 hours. Colony-forming units (CFU) on each plate were enumerated and the number of CFU was reported per cm^2^ of skin.

#### Diet studies

The high-fat diet (TD.06414; 60% of total calories from fat) and the corresponding control diet (TD.150064; 10% of total calories from fat) were purchased from Envigo Teklad Diets. Mice were placed under those diet regimens at weaning (3 weeks old) for 6 weeks before topical association with *S. epidermidis*. Ear thickness was measured with a digital caliper (Mitutoyo). For each mouse, the ear-skin thickness value at a defined time point was calculated by averaging the thickness of both ears measured at this time point. The change in ear-skin thickness over time was reported as the difference related to the first day the mice were first given specific diets.

#### Imiquimod treatment

8-12-week-old C57BL/6J mice were treated daily for 5 consecutive days on each ear pinnae with 10 mg of 5% imiquimod cream (Aldara Cream 5%).

#### Murine tissue processing

Cells from the ear pinnae were isolated as previously described (Naik et al., 2012) Briefly, ears were excised and separated into ventral and dorsal sheets. Ear pinnae were digested in digestion media (RPMI 1640 media supplemented with 2 mM L-glutamine, 1 mM sodium pyruvate and nonessential amino acids, 55 mM β-mercaptoethanol, 20 mM HEPES, 100 U/ml penicillin, 100 μg/ml streptomycin and 0.25 mg/ml Liberase TL purified enzyme blend, Roche), and incubated for 2 hours at 37°C and 5% CO2. Digested skin sheets were homogenized using the Medicon/Medimachine tissue homogenizer system (Becton Dickinson). To separate epidermis from dermis, ear pinnae were first digested for 45 min with 500 CU Dispase (Becton Dickinson) in HBSS without calcium and magnesium. Epidermis was then peeled from dermis with curved forceps, washed in PBS and successively cut with scissors, digested with Liberase TL digestion media for 1 h at 37°C and 5% CO2, homogenized by pipetting up and down, and finally filtered through 70-μm cell strainer.

#### *In vitro* lymphocyte restimulation

For detection of basal cytokine production potential, single- cell suspensions from ear pinnae skin were cultured *ex vivo* for 2.5 hours at 37°C in 5% CO_2_ in complete medium (RPMI 1640 supplemented with 10% fetal bovine serum, 2 mM L-glutamine, 1 mM sodium pyruvate, 1 mM nonessential amino acids, 20 mM HEPES, 100 U/ml penicillin, 100 μg/ ml streptomycin, and 50 mM β-mercaptoethanol) containing 50 ng/ml of phorbol myristate acetate (PMA) (Sigma-Aldrich) and 5 μg/ml of ionomycin (Sigma-Aldrich), and a 1:1000 dilution of brefeldin A (GolgiPlug, BD Biosciences).

#### Primary keratinocytes culture

Primary neonatal mouse keratinocytes were isolated from newborn pups as described previously (Lichti et al., 2008). In brief, 2-3 days old pups were sterilized thoroughly with betadine followed by washing with 70% ethanol. Their skin was stripped off and floated on 0.25% trypsin (GIBCO, Grand Island, NY, USA) overnight (16–18 h) at 4 °C. Epidermal layer was separated from dermis and chopped in medium containing 8% FBS and 1.3 mM calcium (high calcium (HiCa) medium). The cells were filtered through 100 μm cell strainer (Corning Falcon) and seeded in 24-wells plates in medium containing 0.05 mM calcium and 8% Chelex-treated FBS (low calcium (LoCa) medium) at 37 °C in a 7% CO_2_ atmosphere. After 18 h, loosely attached or floating rounded cells and cell aggregates, representing suprabasal and cornifying cells, were removed by washing with PBS. Culture media was changed every 2 days. By day 4 after plating, cells were collected and stimulated with *S. epidermidis* (MOI of 100), LPS (1 μg/ml), R848 (5 μg/ml), LTA (10 μg/ml) for 24 hours.

#### Tetramer-based cell enrichment

f-MIIINA:H2-M3 specific CD8^+^ T cells from spleen were subjected to magnetic bead-based enrichment. In brief, spleen cells were prepared to a single cell suspension and stained for 1 hour at room temperature in the dark with f-MIIINA:H2-M3-streptavidin-phycoerythrin (PE) tetramer in complete RPMI and in the presence of anti-mouse CD16/32 and rat IgG. Samples were then incubated with StemCell anti-PE antibody cocktail (Stem Cell) for 15 minutes at room temperature in the dark and then incubated with StemCell Easysep Magnetic Particles for 10 minutes at room temperature in the dark. Samples were then enriched using a StemCell EasySep Magnet Multistand (StemCell).

#### Flow cytometric analysis

Single-cell suspensions were incubated with combinations of fluorophore-conjugated antibodies against the following surface markers: CD3e (145-2C11), CD4 (RM4-5), CD8b (53- 6.7), CD45 (30-F11), TCRβ (H57-597), MHCII (M5/114.15.2), CD49f (eBioGoH3), CD90.2 (53–2.1), CD31 (MEC13.3), CD34 (RAM34), Sca-1 (D7) and γδTCR (eBioGL3) in Hank’s buffered salt solution (HBSS) for 30 min at 4°C and then washed. LIVE/DEAD Fixable Blue Dead Cell Stain Kit (Invitrogen Life Technologies) was used to exclude dead cells. Cells were then fixed and permeabilized with the Foxp3/Transcription Factor Staining Buffer Set (eBioscience) and stained with fluorophore-conjugated antibodies for at least 60 min at 4°C. For transcription factor (Foxp3), Ki-67 and intracellular cytokine staining, cells were stained with fluorophore-conjugated antibodies against Foxp3 (FJK-16 s), Ki-67 (16A8), IFN-γ (XMG-1.2), and IL-17A (eBio17B7) for 60 min at 4°C. For MAIT cells or f-MIIINA:H2-M3 staining, cells were stained in RPMI complete media for 1 hour at room temperature with 5-OP-RU mMR1 tetramer or f-MIIINA:H2-M3 tetramer (provided by the NIH Tetramer Core Facility). Each staining was performed in the presence of purified anti-mouse CD16/32 (clone 93) and purified rat gamma globulin (Jackson Immuno- research). All antibodies were purchased from eBioscience, Biolegend, BD Biosciences, or Miltenyi Biotec. Ecotropic and non-ecotropic ERV envelope protein (MLV SU) was detected using the 83A25 monoclonal antibody (Evans et al., 1990). Cells were incubated with hybridoma 83A25 supernatant or rat IgG2a isotype control, followed by staining with a biotinylated anti-rat IgG2a antibody (clone RG7/1.30, BD Biosciences), and streptavidin-PE-Cy7 (BioLegend). For MLV SU expression analysis, the iMFI, which reflects the total functional response and is calculated by multiplying the frequency by the MFI (Darrah et al., 2007), was used. Cell acquisition was performed on a BD Fortessa X-20 flow cytometer using FACSDiVa software (BD Biosciences) and data were analyzed using FlowJo software (TreeStar).

#### Keratinocyte purification

Interfollicular epidermal keratinocytes were purified by cell sorting from the epidermis of ear pinnae from unassociated or *S. epidermidis*-associated mice treated or not with antiretroviral drugs (when indicated). Mice were daily associated for 7 days (for bulk RNA-sequencing) or every other day a total of four times (for single-cell RNA sequencing), and the experiments were performed at day 7 or 14 post association, respectively. Cell suspensions obtained from the epidermis of ear pinnae were incubated with the following antibodies: anti-CD16/32 (93), anti-CD31 (MEC13.3), anti-CD34 (RAM34), anti-CD45 (30-F11), anti-CD49f (eBioGoH3), anti-Sca-1 (D7) in the presence of DAPI. Interfollicular epidermal keratinocytes were sorted by flow cytometry on a FACSAria (BD Biosciences) as DAPI^–^CD45^–^CD31^–^CD34^–^CD49f^+^Sca-1^+^cells.

#### RNA-Seq Library preparation and gene analysis

Keratinocyte RNA was extracted using the RNEasy Micro Kit (QIAGEN). RNA-seq libraries were prepared using Illumina TruSeq Stranded Total RNA Library Prep for paired-end sequencing and sequenced on a HiSeq4000. Sequencing reads were mapped to the C57BL/6 mouse genome (GRCm38: mm10) with STAR using default parameters (Dobin et al., 2013). Gene expression was assessed using HOMER 4.11 (Heinz et al., 2010), specifically the analyzeRepeats function with parameters mm10, -count exons -condenseGenes -noadj, and differential expression was calculated with DESeq2 (Love et al., 2014). Principal component analysis (PCA) was done with R. Differentially expressed genes were defined as genes that had a False Discovery Rate (FDR) < 0.05 and Fold Change (FC) > 2. Gene ontology analysis was done with metascape (Zhou et al., 2019). Sequencing data is deposited in the NCBI SRA under PRJNA664825.

#### RNA-Seq retroelement analysis

Sequencing reads were mapped to the appropriate host genome (C57BL/6 mouse genome (GRCm38: mm10) or *Homo sapiens* (hg38)) with STAR using stringent mapping parameters such that reads aligned to multiple regions of the genome were considered unmapped (–outFilterMultimapNmax 2). Retroelement expression was assessed using HOMER 4.11 by defining retroelements using the annotation provided by the Genome-based Endogenous Viral Element Database (gEVE) for GRCm38 or GRCh38 where appropriate (Nakagawa and Takahashi, 2016). The gEVE database identifies specific chromosomal regions as expressing a specific ORF; these chromosomal coordinates were directly used in HOMER to specify regions of the genome that encoded a retroelement. Reads were analyzed using the analyzeRepeats function with parameters mm10, -count genes -noadj, and differential expression was calculated with DESeq2. Differentially expressed retroelements were defined as loci that had a False Discovery Rate (FDR) < 0.05.

#### RNA Extraction, cDNA Synthesis, and qPCR of skin tissue

A 4 mm biopsy punch was collected from the center of ear pinnae and submerged in RNAlater (Sigma) and stored at −80°C. Total tissue RNA was isolated from skin tissue using the RNeasy Fibrous Tissue Mini Kit (QIAGEN), as per manufacturer’s instructions. DNase-treated total RNA (1 mg) was reverse transcribed into cDNA with Omniscript RT kit (QIAGEN) following manufacturer’s instructions. qPCR was performed using the iQ SYBR Green Supermix (Life Technologies) on a QuantStudio 6 Flex Real-Time PCR (Life Technologies) using the previously published primer sequences listed (Table S1; Collins et al., 2015; Karimi et al., 2011; Macfarlan et al., 2011; Stoltz et al., 2019; Yin et al., 2011; Yoshinobu et al., 2009; Young et al., 2012b). The housekeeping gene *Gapdh* was used to normalize the Critical Threshold (CT) values. Analysis was conducted with the ΔCT method, using the algorithm Value = 2^(CT value of^ ^*Gapdh*^ ^– CT value of target)^ x 10^4^. The data are represented as fold increase of *S. epidermidis*-associated relative to unassociated mice.

#### BLAST search for Reverse Transcriptase Homologs

Amino acid sequences for ltrA related to Group II Introns in other Staphylococcus members (NCBI accession numbers: WP_115926208.1, WP_015728874.1) as well as a representative RT-Cas1 gene (WP_013659858.1) were aligned to the *Staphylococcus epidermidi*s NIHLM087 genome using tBLASTn with an e-value cutoff of 0.1.

#### Identification of retroelement loci encoding potentially active RT

Murine ERV loci encoding potential active RT were identified using a prediction model based on conservation of active site amino acid residues. The following motifs in the RVT_1 domain of RTs (http://pfam.xfam.org/family/PF00078) were used: YxDD and DLKDAF, involved in Mg^2+^ ion coordination and polymerization site; [WY]xxxLPQG and QDLREVNK involved in template binding and polymerization site. All motifs were found to be conserved in the active RTs of HIV-1, GLN2, MLV, MMTV and HERVK and motif YxDD was additionally conserved in LINE-1 ORF2p. Template binding motifs were further validated based on the reported structure of HIV-1 RT (PDB: 1RTD) (Huang et al., 1998).(Murine LINE-1 loci encoding potential active RT were identified based on existing annotation for the presence of an intact ORF2p in L1base (http://l1base.charite.de; Penzkofer et al., 2017).

#### Single-cell RNA sequencing

WT mice (n = 5 per group) were unassociated or *S. epidermidis*-associated and treated with vehicle or antiretroviral drugs by oral gavage, beginning at 1 week before *S. epidermidis* association for a total of 3 weeks. At day 14 post association mice ears was collected and digested as described above to separate epidermis from dermis. Isolated cells were stained with antibodies against surface markers and TotalSeq-A hashtag oligonucleotide (HTO) antibodies (BioLegend) (Table S2). Hash tags with not enough reads (due to insufficient staining) were excluded from the downstream analysis. Interfollicular epidermis keratinocytes were sorted as live (DAPI^−^) CD31^−^CD45^−^CD34^−^Sca-1^+^CD49f^+^. The Chromium Single Cell Controller (10X Genomics) was overloaded with 45,000 cells (3,000 cells per sample) per lane and the library was prepared following the 10X Genomics guidelines. The hashtag library was processed according to guidelines from Stoeckius et al. (2018). A total of 4 lanes were prepared and libraries were sequenced on an Illumina Nextseq500 (Next Seq 500/550 High Output Kit v2, Illumina). For processing, cellranger 4.0.0 (10X Genomics) was used to map the raw sequencing data to the mm10 reference genome and HTO and mRNA libraries were processed together with cellranger count. Only singlet cells with less than 10% mitochondrial contamination were used for downstream analysis, leaving 26,646 cells. Downstream analysis was performed using Seurat version 3.1.4. Data was normalized, scaled, principal components analysis was performed, and neighbors were found using 11 dimensions. Uniform manifold approximation and projection (UMAP) reduction was performed on this dataset with 25 dimensions. Clusters were assigned using marker genes defined in earlier studies on epidermal keratinocytes (Joost et al., 2016). Differentially expressed genes between treatment groups were identified running FindMarkers using the non-parameteric Wilcoxon rank sum test between each treatment. To identify differentially expressed genes between clusters FindAllMarkers were run. P values were adjusted using a Bonferroni correction.

#### Immunofluorescence/confocal microscopy of ear pinnae

Ear pinnae were split with forceps, fixed in 1% paraformaldehyde solution (Electron Microscopy Sciences) overnight at 4°C and blocked in 1% BSA, 0.25% Triton X blocking buffer for 2 hours at room temperature. Tissues were first stained with anti-CD4 (RM4-5, eBioscience), anti-CD8α (clone 53- 6.7, eBioscience), anti-CD49f (GoH3, eBioscience) antibodies overnight at 4°C, washed three times with PBS and then mounted with ProLong Gold (Molecular Probes) antifade reagent. Ear pinnae images were captured on a Leica TCS SP8 confocal microscope with a 40X oil objective (HC PL APO 40X/1.3 oil). Images were analyzed using Imaris Bitplane software.

#### Histology

Mice were euthanized 14 days after topical application of *S. epidermidis.* Unassociated mice were used as controls. The ears from each mouse were removed and fixed in PBS containing 10% formalin. Paraffin-embedded sections were cut at 0.5 μm, stained with hematoxylin and eosin, and examined histologically.

### Quantification and statistical analysis

Groups were compared with Prism V7.0 software (GraphPad) using the two-tailed unpaired Student’s t test, one-way analysis of variance (ANOVA) with Holm-Šidák multiple-comparison test, or two-way ANOVA with Holm-Šidák multiple-comparison test where appropriate. Differences were considered to be statistically significant when p ≤ 0.05.

### Additional resources

This study did not generate any additional resources.
