# Supplementary material for: Endogenous retroviruses promote homeostatic and inflammatory responses to the microbiota
Source: Cell. 2021 Jul 8;184(14):3794–3811.e19. doi: 10.1016/j.cell.2021.05.020 (PMC8381240; doi:10.1016/j.cell.2021.05.020)
Supplement: Document S1. Tables S1 and S2 [file mmc1.pdf]

Cell, Volume *184*

Supplemental information

Endogenous retroviruses promote homeostatic

and inflammatory responses to the microbiota

Djalma S. Lima-Junior, Siddharth R. Krishnamurthy, Nicolas Bouladoux, Nicholas Collins, Seong-Ji Han, Erin Y. Chen, Michael G. Constantinides, Verena M. Link, Ai Ing Lim, Michel Enamorado, Christophe Cataisson, Louis Gil, Indira Rao, Taylor K. Farley, Galina Koroleva, Jan Attig, Stuart H. Yuspa, Michael A. Fischbach, George Kassiotis, and Yasmine Belkaid

Table S1: Primer sequences, related to STAR Methods.

Oligo Name	Sequence (5' to 3')	Reference
eMLV *env* F	CCAGGGACCACCGACCCACCG	Young, Ploquin et al., 2012
eMLV *env* R	TAGTCGGTCCCGGTAGGCCTCG	Young, Ploquin et al., 2012
eMLV gag/pol F	CACTTTGAGGGATCAGGAGCC	Young et al., 2012
eMLV gag/pol R	CTTCTAGGTTTAGGGTCAACACCTGT	Young et al., 2012
MMTV spliced F	AGAGCGGAACGGACTCACCA	Young et al., 2012
MMTV spliced R	TCAGTGAAAGGTCGGATGAA	Young et al., 2012
MMTV F	GACATGAAACAACAGGTACATGA	Yin et al., 2011
MMTV R	GGACTGTTGCAAGTTTACTC	Yin et al., 2011
xMLV F	TCTATGGTACCTGGGGCTC	Yoshinobu et al., 2009
xMLV R	GGCAGAGGTATGGTTGGAGTAG	Yoshinobu et al., 2009
pMLV/mpMLV common F	CCGCCAGGTCCTCAATATAG	Yoshinobu et al., 2009
pMLV R	AGAAGGTGGGGCAGTCT	Yoshinobu et al., 2009
mpMLV R	CGTCCCAGGTTGATAGAGG	Yoshinobu et al., 2009
GLN F	CGTAAGGACCCTAGTGGCTG	Karimi et al., 2011
GLN R	GCACTCACTCTTCTTCACTCTG	Karimi et al., 2011
IAP F	AAGCAGCAATCACCCACTTTGG	Collins et al., 2015
IAP R	CAATCATTAGATGCGGCTGCCAAG	Collins et al., 2015
MMERVK F	CAAATAGCCCTACCATATGTCAG	Stoltz et al., 2019
MMERVK R	GTATACTTTCTTCTTCAGGTCCAC	Stoltz et al., 2019
MaLR (MTA) F	ATGTTTTGGGGAGGACTGTG	Karimi et al., 2011
MaLR (MTA) R	AGCCCCAGCTAACCAGAAC	Karimi et al., 2011
MusD/EnTII common F	GTGCTAACCCAACGCTGGTTC	Karimi et al., 2011
MusD R	CTCTGGCCTGAAACAACTCCTG	Karimi et al., 2011
ETnII R	ACTGGGGCAATCCGCCTATTC	Karimi et al., 2011
MervI Pol F	ATCTCCTGGCACCTGGTATG	Macfarlan et al., 2011
MervI Pol R	AGAAGAAGGCATTTGCCAGA	Macfarlan et al., 2011
Gapdh F	AGGCTCAAGGGCTTTTAAGG	
Gapdh R	ATCCTGTAGGCCAGGTGATG	

Table S2: HTO Hasing index, related to STAR Methods.

Samples	Treatment	Sorted population	HTO	Barcode Sequence
Mouse1	Control	Keratinocyte	A301	ACCCACCAGTAAGAC
Mouse2	Control	Keratinocyte	A302	GGTCGAGAGCATTCA
Mouse3	Control	Keratinocyte	A303	CTTGCCGCATGTCAT
Mouse4	Control	Keratinocyte	A304	AAAGCATTCTTCACG
Mouse5	Control	Keratinocyte	A0305	CTTTGTCTTTGTGAG
Mouse6	Sepi	Keratinocyte	A0306	TATGCTGCCACGGTA
Mouse7	Sepi	Keratinocyte	A0307	GAGTCTGCCAGTATC
Mouse8	Sepi	Keratinocyte	A0308	TATAGAACGCCAGGC
Mouse9	Sepi	Keratinocyte	A0309	TGCCTATGAAACAAG
Mouse10	Sepi	Keratinocyte	A0310	CCGATTGTAACAGAC
Mouse11	Sepi_antiRT	Keratinocyte	A0311	GCTTACCGAATTAAC
Mouse12	Sepi_antiRT	Keratinocyte	A0312	CTGCAAATATAACGG
Mouse13	Sepi_antiRT	Keratinocyte	A0313	CTACATTGCGATTTG
Mouse14	Sepi_antiRT	Keratinocyte	A0314	CTTTCGCCAACTCTG
Mouse15	Sepi_antiRT	Keratinocyte	A0315	TGCCTATGAAACAAG
